# Integrated Metabolome and Transcriptome Analyses Reveal Amino Acid Biosynthesis Mechanisms during the Physiological Maturity of Grains in Yunnan Hulled Wheat (*Triticum aestivum* ssp. *yunnanense* King)

**DOI:** 10.3390/ijms241713475

**Published:** 2023-08-30

**Authors:** Chuanli Zhang, Ping Zhang, Xuesong Zhang, Qianchao Wang, Junna Liu, Li Li, Shunhe Cheng, Peng Qin

**Affiliations:** 1 College of Agronomy and Biotechnology, Yunnan Agricultural University, Kunming 650201, China; 2007078@ynau.edu.cn (C.Z.); 2021110031@stu.ynau.edu.cn (P.Z.); 2020240160@stu.ynau.edu.cn (X.Z.); 2020110028@stu.ynau.edu.cn (Q.W.); 2021110026@stu.ynau.edu.cn (J.L.); 2019210130@stu.ynau.edu.cn (L.L.); 2 College of Tropical Crops, Yunnan Agricultural University, Kunming 650201, China

**Keywords:** Yunnan hulled wheat, metabolomics, transcriptomics, waxy grain, mature grains

## Abstract

Yunnan hulled wheat (YHW) possesses excellent nutritional characteristics; however, the precise amino acid (AA) composition, contents, and molecular mechanisms underlying AA biosynthesis in YHW grains remain unclear. In this study, we aimed to perform metabolomic and transcriptomic profiling to identify the composition and genetic factors regulating AA biosynthesis during the physiological maturation of grains of two YHW genotypes, Yunmai and Dikemail, with high and low grain protein contents, respectively. A total of 40 and 14 differentially accumulated amino acids (AAs) or AA derivatives were identified between the waxy grain (WG) and mature grain (MG) phenological stages of Yunmai and Dikemail, respectively. The AA composition differed between WG and MG, and the abundance of AAs—especially that of essential AAs—was significantly higher in WG than in MG (only 38.74–58.26% of WG). Transcriptome analysis revealed differential regulation of structural genes associated with the relatively higher accumulation of AAs in WG. Weighted gene co-expression network analysis and correlation analyses of WG and MG indicated differences in the expression of clusters of genes encoding both upstream elements of AA biosynthesis and enzymes that are directly involved in AA synthesis. The expression of these genes directly impacted the synthesis of various AAs. Together, these results contribute to our understanding of the mechanism of AA biosynthesis during the different developmental stages of grains and provide a foundation for further research to improve the nutritional value of wheat products.

## 1. Introduction

Amino acids (AAs) in grains of cereal crops are important nutritional components for humans [1,2] and are basic nutrients that are available in foods [3]. Today, malnourished people account for 30% of the world’s population because of insufficient intake of AAs. Therefore, improving the amino acid (AA) contents of cereal crops is an effective way to meet nutritional needs [4,5]. AAs are usually obtained from plant-derived foods and determine the nutrient quality of cereal grains and the human diet [6,7]. Wheat (*Triticum aestivum*) is a major cultivated cereal worldwide [8] and feeds two-fifths of the world’s population [9,10]. However, wheat alone cannot satisfy the daily dietary nutritional needs of humans, because its AA content is insufficient [11,12]. Hence, it is important to understand the accumulation and composition of AAs, and particularly the regulation of their biosynthesis, to improve the nutrient quality of wheat grains. Yunnan hulled wheat (YHW; *T. aestivum* ssp. *yunnanense* King), a unique, primitive, semi-wild hexaploid wheat, was discovered in Yunnan Province in Southwest China [13] and is one of the three Chinese endemic common wheat subspecies [14]. It has the advantage of good quality, particularly due to its high protein content, which can reach up to 18.24% [14,15]. Overall, the protein content ranges from 10 to 18% in wheat grains [16,17]. Therefore, it is considered to be one of the most valuable resources in terms of improving the nutritional quality of wheat.

Proteins are indispensable active macromolecules that are involved in biological processes [18]. AAs are the basic units of proteins and substrates of protein synthesis [19]. There are usually 20 main AAs in cereal grains, including 9 essential amino acids (EAAs), namely, phenylalanine (Phe), lysine (Lys), leucine (Leu), threonine (Thr), isoleucine (Ile), tryptophan (Trp), methionine (Met), valine (Val), and histidine (His), which humans cannot synthesize [20,21]. Lys, Thr, Met, and Ile are the major AAs in wheat grains [22]. The lack of one of these four AAs in humans markedly decreases the assimilation and utilization of other AAs and may lead to severe diseases [23,24,25]. Lys and Thr widely participate in various biological activities in humans [26]. For example, Lys can accelerate protein assimilation and utilization and improve neurological and immune system functions [23], and insufficient Lys intake can reduce human defense against viruses [27,28]. Thr is a critical AA that regulates the intestinal immune response and synthesizes mucosal proteins [29], and its deficiency can result in mental disorders, indigestion, and fatty liver disease [30]. Therefore, appropriate supplementation of proteins and EAAs is extremely important for regulating human nutrition and health. 

AAs are important functional nutrients in cereal grains and are indispensable molecules in many biochemical synthesis pathways [31]. AAs are fundamental constituents of proteins and are precursors of other secondary metabolites [32]. Grain protein content (GPC) and various amino acid content (AAC), especially essential amino acid content (EAAC), are key indicators for evaluating the nutritional value of crop grains. Previous studies have shown that AA biosynthesis has been extensively studied in microorganisms, and some key genes regulating AAC have been identified from *Arabidopsis*, tobacco, maize, soybean, rice, rapeseed, and so on [33]. However, there is still a need for a comprehensive study on the variations in AAC and AA components at different stages of grain development. Additionally, further investigation is required to understand the relationship between AAC and components at different stages of development, as well as the mechanisms underlying differences in biosynthesis. Moreover, research on the composition and AAC, as well as their biosynthetic mechanisms at different stages of wheat grain development (especially in YHW), has rarely been conducted. AA composition regulates plants’ growth, development, and nutrient quality significantly. The composition and concentration of AAs are often influenced by pesticides used in wheat protection. For example, the herbicide sulfonylurea combined with the fungicides spiroketalamine and triazole reduced the concentration of AAs, while a biostimulator based on nitrophenols increased the levels of AAs [34,35]. The composition and AAC in wheat grains are also influenced by many other key factors [36], including developmental stages. Wheat grain development can be divided into three stages: enlargement, filling, and physiological maturity [37]. Among these, grain filling and physiological maturity are key phases for nutrient accumulation [38], which determines the quality of grains. The final harvested wheat product is the grain, and the waxy and mature stages of physiological maturity are the wheat harvest periods. The timing of harvest can directly affect the yield and quality of wheat grains. However, there is little knowledge about the relationship between the AA composition and content and the different developmental stages of wheat’s physiological maturity, as well as the biosynthesis mechanisms and metabolic regulation. Therefore, the comprehensive analysis reveals the regulatory mechanisms of AA biosynthesis at the wax and mature stages that lay the foundation for improving the AAC, especially the EAAC and nutritional quality of wheat grains.

Previous studies have investigated the changes in transcription and metabolic networks at the physiological maturity stage of wheat grains. However, the changes in AAC and composition in the transition from waxy to mature stages of wheat grains have not been studied. In this study, we took an integrated approach—widely targeted metabolomics and transcriptomics—to examine differences in AA species and content, as well as to analyze AA biosynthesis between the waxy grain (WG) and mature grain (MG) stages of physiological maturity in Yunnan hulled wheat. The differences in contents and synthetic pathways of AAs between the WG and MG stages in two YHW lines were analyzed to reveal the biosynthesis rule of AAs in MG Yunnan hulled wheat. This investigation provides brand-new perspectives on the changes in metabolic and transcriptional regulatory networks of amino acids content, composition, and biosynthesis during the waxy and mature stages of YHW grains. It also preliminarily elucidates the optimal harvest period of wheat from the perspective of transcription and metabolism, which could improve wheat quality distinctly.

## 2. Results

### 2.1. Plant Material and Physiological Maturity Selection of Two YHW Lines

**Figure 1:**
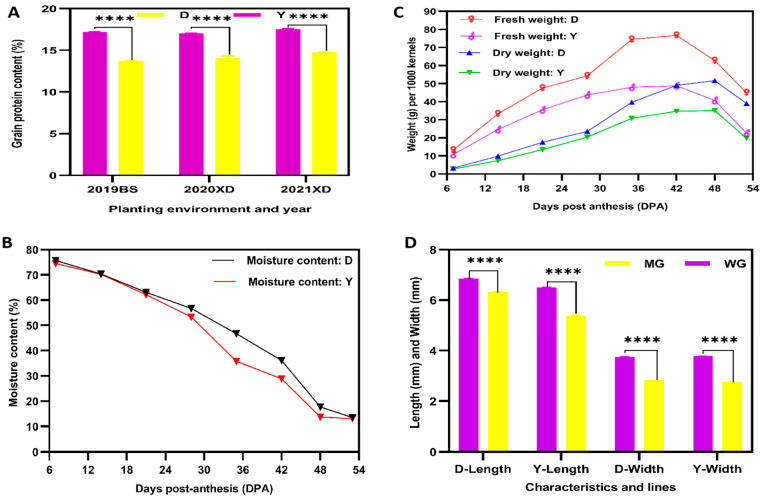
Comparisons of the characteristics of two Yunnan hulled wheat lines grains: Dikemai1 (D) and Yunmai 0606 (Y). (**A**) Protein contents in D and Y maturation grains grown in different environments in different years: Baoshan (BS; the wheat was cultivated in the field in 2019) and Xundian (XD; the wheat was cultivated in the field in 2020 and was cultivated in a greenhouse in 2021). (**B**) Changes in fresh and dry weights of the two grain lines over the course of development. (**C**) Changes in the moisture content of developing grains. (**D**) Comparison of grain length and width between the waxy grain (WG) and mature grain (MG) developmental stages of the two grain lines. The statistical test for significant differences was a post hoc test of multiple comparisons with analysis of variance (ANOVA). Values are presented as means ± standard errors (SE) (n = 3), and bars indicate the SE; **** *p* < 0.0001.

From a pool of fifty YHW lines originating in Yunnan, China, two lines, Dikemai1 (D; low GPC) and Yunmai 0606 (Y; high GPC), were selected for analyses. The two grains were grown in two different environments over three consecutive years: Baoshan (2019) and Xundian (2020 and 2021). Significant differences in the total GPC of the two lines were observed for all three years (Figure 1A). Whole YHW grains were sampled from 7 to 53 days post-anthesis (DPA), and the characteristics of the developing grains were recorded. During this period, the moisture content of the grains of both lines gradually decreased to approximately 13% at 53 DPA (Figure 1B). The fresh and dry weight gradually increased, reaching maximum levels at 42 and 48 days, respectively, after which a decrease was observed (Figure 1C). These results show that the developing grains entered the WG and MG stages at 42 and 53 DPA, respectively. In both lines, significant decreases in grain length and width from the WG to the MG stage were observed (Figure 1D). Therefore, grains from these two YHW lines at 42 and 53 DPA, respectively, were selected for physiological, metabolomic, and transcriptomic analyses. The complete set of recorded results can be found in Tables S1 and S2.

### 2.2. Phenotype and Total Amino Acid Contents of D and Y

**Figure 2:**
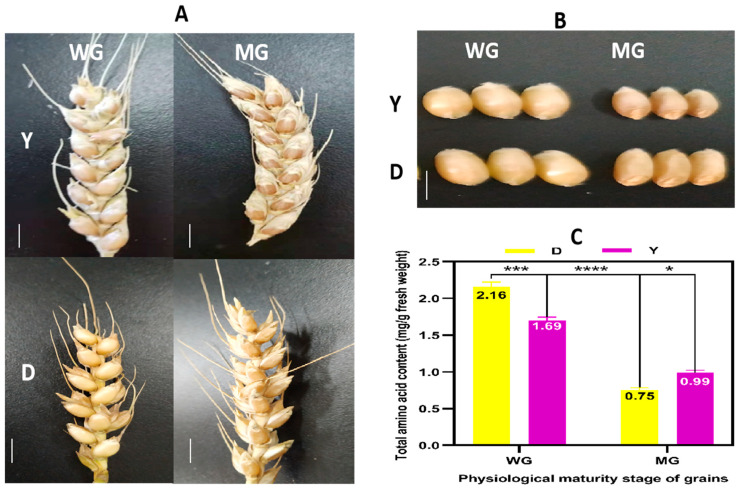
Changes in Yunnan hulled wheat (YHW) grains between the waxy grain (WG) and mature grain (MG) stages. The (**A**) spikes and (**B**) grains of YHW lines Yunmai 0606 (Y) and Dikemai1 (D) at the WG and MG stages. Scale bars: the wheat spikes and wheat grains, 1 cm. (**C**) Total amino acid contents of grains of Y and D at the WG and MG stages. The statistical test for significant differences was a post hoc test of multiple comparisons with analysis of variance (ANOVA). Values are presented as means ± standard errors (SE) (n = 3), and bars indicate the SE; * *p* < 0.05; *** *p* < 0.001; **** *p* < 0.0001.

The phenotypes of D and Y spikes and grains were recorded at the WG and MG stages. The brown grain color of both lines was similar and did not change much during the physiological maturation process (Figure 2A,B). To understand the difference in GPC observed between D and Y, the total amino acid content (TAAC) of WG and MG of the two lines was measured, and significant differences between the two lines, as well as between the two developmental stages, were found (Figure 2C). The TAAC of YMG was more than 1.3 times that of DMG, confirming the difference in GPC determined using near-infrared analysis. Compared with that of MG, the TAAC for WG of D and Y was significantly increased, with fold changes of 2.8 and 1.7, respectively, reaching a peak of 2.16 mg/g fresh weight (Table S3). To elucidate the molecular mechanisms underlying the variation in AA accumulation during the physiological maturation of YHW grains, samples were subjected to metabolomic and transcriptomic analyses.

### 2.3. Metabolome Analyses of AAs and Derivatives in WG and MG of Y and D

**Figure 3:**
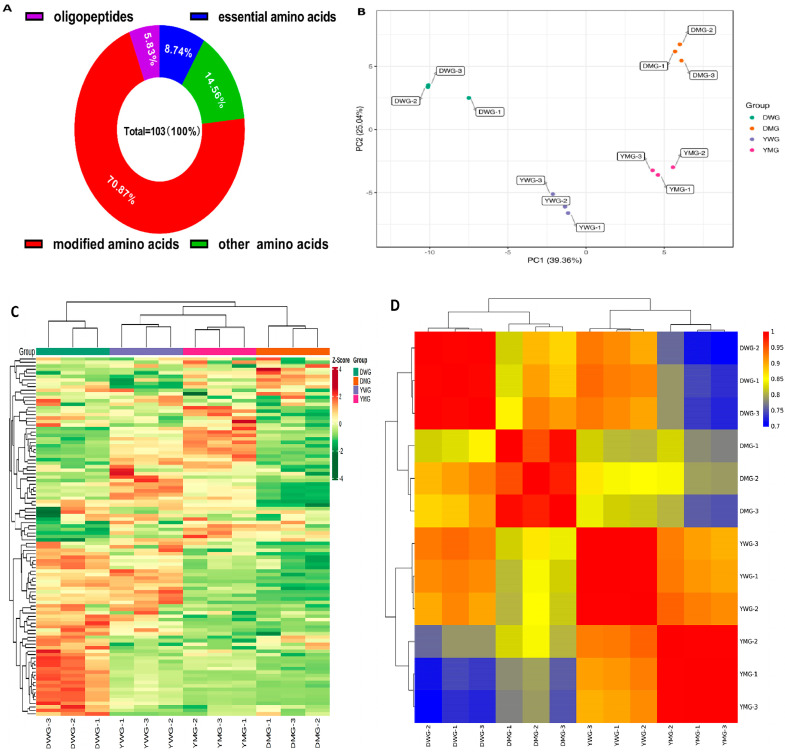
Metabolome analyses of amino acids and derivative compounds (AADCs) in Yunnan hulled wheat: (**A**) Composition of the identified AADCs. (**B**) Principal component analysis (PCA) of the AADCs of two wheat lines, Yunmai 0606 (Y) and Dikemai1. (D), at the waxy grain (WG) and mature grain (MG) developmental stages. (**C**) Heatmap of the hierarchical cluster analysis (HCA) conducted on the metabolic compounds in DWG (green panel), DMG (orange panel), YWG (purple panel), and YMG (pink panel). A red color indicates a relatively higher amount of each compound, while a green color indicates a relatively lower amount of each compound. (**D**) Heatmap of the correlation analysis. Red indicates a high similarity, while blue indicates lower similarity.

A total of 103 AAs and derivative compounds (AADCs) were identified (Table 1) in 12 samples (3 each of DWG, DMG, YWG, and YMG): 9 EAAs (8.74%), 15 other AAs (14.56%), 73 modified AAs (70.87%), and 6 oligopeptides (5.83%). Pathway analysis using the Kyoto Encyclopedia of Genes and Genomes (KEGG) database enabled the annotation of 48 AADCs (Figure 3A and Table S4). A principal component analysis (PCA) revealed distinct separations between the four groups (DWG, DMG, YWG, and YMG), and 39.36% and 25.04% of the total variance could be explained by PCA1 and PCA2, respectively (Figure 3B). Furthermore, the difference in grain AADCs between the two developmental stages (WG and MG) within the same line was greater than that between the two lines (Y and D) at the same developmental stage. Hierarchical cluster analysis (HCA) also delineated four groups, with two primary categories and four main clusters (Figure 3C and Figure S1A). Category I comprised 68 AADCs, including 8 EAAs: Leu, Ile, Phe, Thr, Val, Lys, His, and Met. Category II included 35 AADCs, only one of which (Trp) is an EAA (Figure S1A). The relative abundance of the AADCs exhibited distinct differences between DWG, DMG, YWG, and YMG. Specifically, the relative abundance of AADCs was highest in DWG within cluster 1, followed by YWG in cluster 2, YMG in cluster 3, and DMG in cluster 4 (Figure S1A), corroborating the results of the TAAC and PCA analyses. Furthermore, the correlation analysis indicated significant correlations between biological replicates of the same samples (Figure 3D and Figure S1B). Notably, these results show that the WG and MG of D and Y grains possessed distinct AADCs profiles, indicating dynamic changes in AADCs in the two YHW lines during specific developmental stages. 

An orthogonal partial least squares discriminant analysis (OPLS-DA) was conducted to identify the variables responsible for the differences between the four groups during the physiological maturation of wheat grains. OPLS-DA was utilized to assess the AADC contents of the same line across different periods, as well as those between different lines during the same period. The results of the comparisons were as follows: DWG and DMG (R2X = 0.744, R2Y = 1, Q2 = 0.968), YWG and YMG (R2X = 0.655, R2Y = 1, Q2 = 0.967), DWG and YWG (R2X = 0.692, R2Y = 1, Q2 = 0.936), and DMG and YMG (R2X = 0.709, R2Y = 1, Q2 = 0.946) (Figure S2). The Q2 values of all comparison groups exceeded 0.93, indicating the stability of these models. The OPLS-DA score map demonstrated that DWG, DMG, YWG, and YMG are distinctly separated in pairs, indicating that there are significant differences in the metabolic phenotypes of AADCs between WG and MG of lines D and Y.

### 2.4. Changes in AADCs during Developmental Stages Differed between the Two Wheat Lines

**Figure 4:**
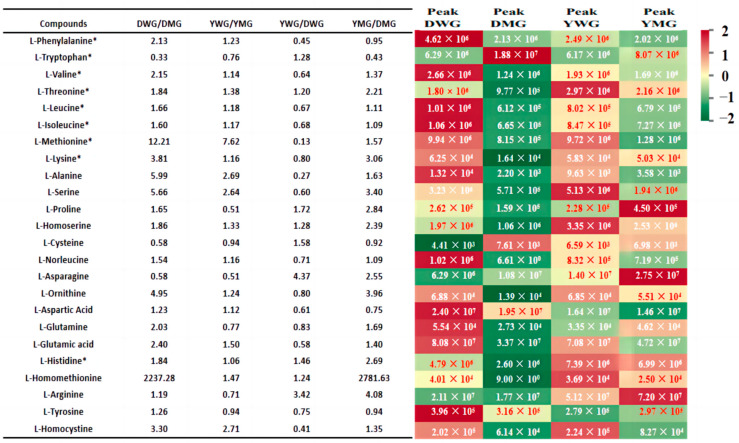
The relative abundances and comparison of conventional amino acids between two Yunnan hulled wheat lines, Dikemai1 (D) and Yunmai 0606 (Y), at two physiological maturation stages, waxy grain (WG) and mature grain (MG). The red color represents a high relative abundance, while the green color represents low relative abundance. For each amino acid, the average peak of the three biological samples of each of the four different groups is provided on the heatmap, and the relative abundance ratios of the four comparison groups are shown in the table. * indicates essential amino acids.

**Figure 5:**
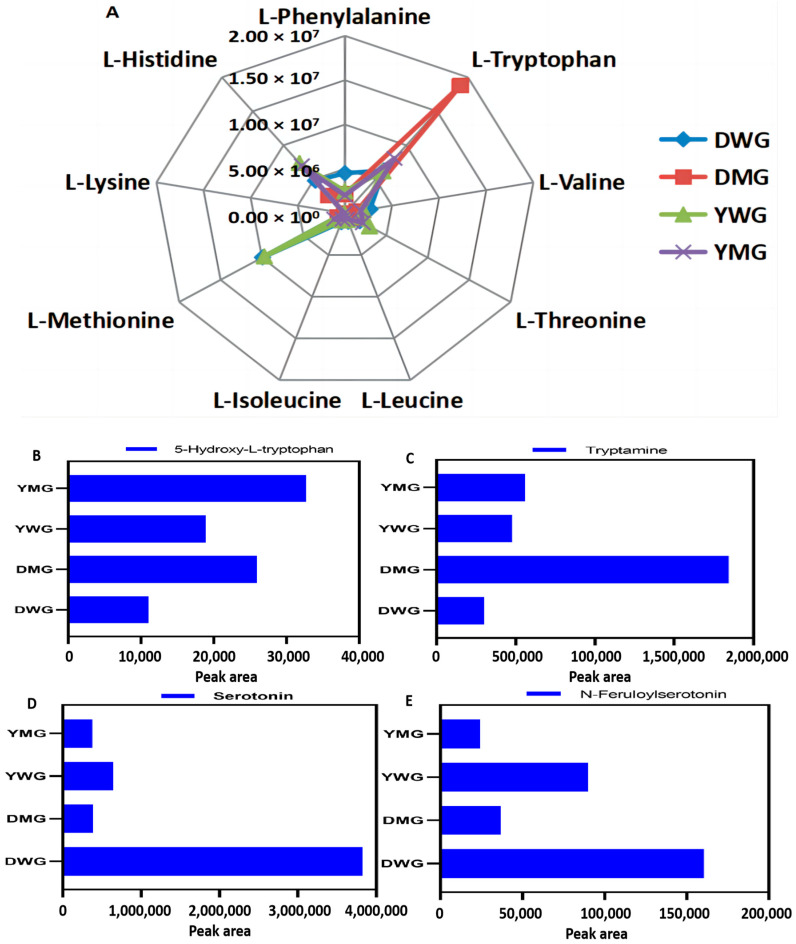
Relative contents radar map of essential amino acids and histogram of four differential tryptophan metabolites among DWG, DMG, YWG, and DMG. (**A**) The radar map of essential amino acids relative contents. (**B**–**E**) The histogram of four differential tryptophan metabolites relative contents among DWG, DMG, YWG, and DMG.

A total of 24 conventional amino acid components (CAACs), comprising 9 EAAs and 15 non-EAAs, were detected. A heatmap depicting the differences in CAACs and a radar map illustrating the AA contents among DWG, DMG, YWG, and YMG were constructed (Figure 4 and Figure 5A). Overall, glutamate (Glu), arginine (Arg), aspartate (Asp), asparagine (Asn), and Trp exhibited the highest levels, while cysteine (Cys) exhibited the lowest. While the composition of conventional amino acids (CAAs) during the different stages of maturation was very similar in both lines, the abundance of the CAAs varied. The abundance of eight of the nine EAAs (all except Trp; fold change was 0.33 or 0.76) was higher in WG (fold change from 1.06 to 12.21) than in MG. With the exception of Cys and Asn (fold change of 0.58), the levels of the non-EAAs (fold change from 1.19 to 2237.28) in DWG were higher than they were in DMG. Except for proline (Pro), tyrosine (Tyr), Asn, glutamine (Gln), Cys, and Arg (fold change from 0.51 to 0.94), the abundance of the other non-EAAs (fold change from 1.12 to 2.71) was higher in YWG than in YMG. With the exception of Trp, Phe, Tyr, Asp, and Cys (fold change from 0.43 to 0.95), the levels of all AAs were higher in YMG than in DMG, with the greatest differences in the abundance of Lys, Met, Val, Leu, Ile, Thr, and His (relative concentrations from 1.64 × 10^4^ to 6.99 × 10^6^). Overall, most CAAs exhibited a decline in their levels from the WG to the MG stage. In general, the abundance of CAAs was higher in Y than in D, and this difference was especially clear in the MG stage. These results corroborated those of the GPC, TAAC, and PC analyses. Furthermore, the contents of metabolites of the tryptophan metabolism pathway differed between the WG and MG stages (Figure 5B–E and Table S5). The levels of tryptamine and 5-hydroxytryptophan, intermediates of the pathway converting Trp to 5-hydroxytryptamine, were lower in WG than in MG. Conversely, serotonin and its derivative, N-feruloserotonin, exhibited higher levels in WG than in MG.

**Figure 6:**
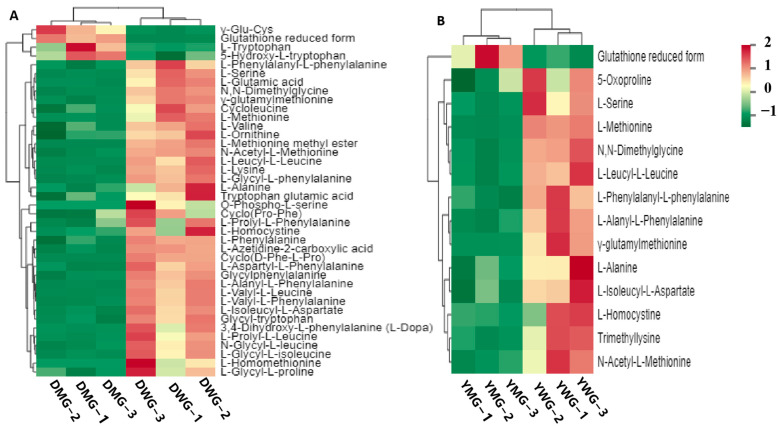

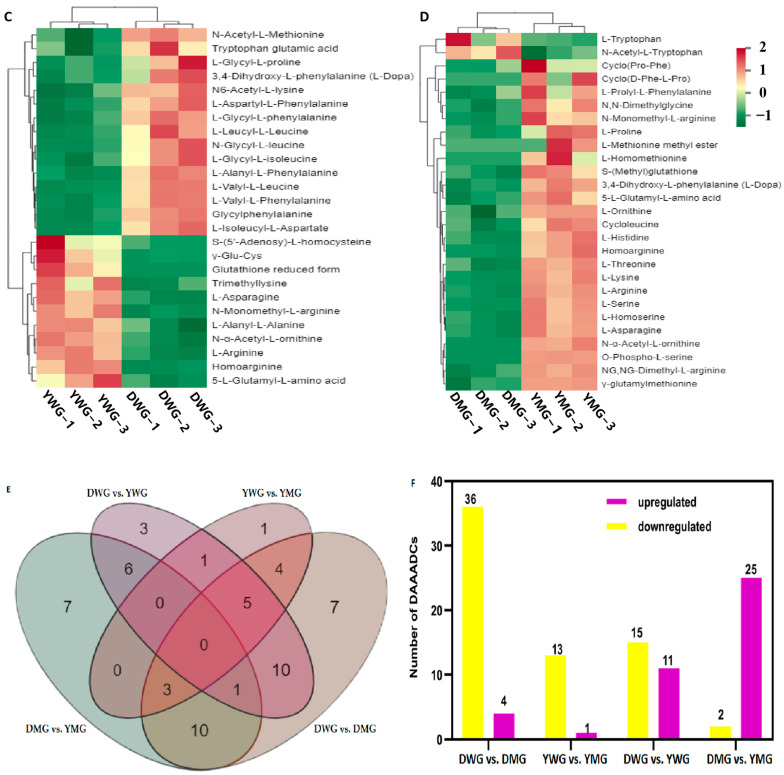
Differentially accumulated amino acids and their derivative compounds (DAAADCs) among two wheat lines Dikemai1 (D) and Yunmai 0606 (Y), at two physiological maturation stages, waxy grain (WG) and mature grain (MG). Heatmap of the DAAADCs in (**A**) DWG vs. DMG, (**B**) YWG vs. YMG, (**C**) DWG vs. YWG, and (**D**) DMG vs. YMG. The color scale shows the relative level of metabolite accumulation, with red indicating a high level and green indicating a low level. (**E**) Venn diagram showing the number of DAAADCs among the different comparison groups. (**F**) The numbers of DAAADCs that were upregulated or downregulated among the different comparison groups.

Among DWG, DMG, YWG, and YMG, differential accumulation of 58 AADCs was observed (Table S6). A k-means analysis divided the 58 differentially accumulated AADCs (DAAADCs) into 10 subclasses, and the relative contents of the DAAADCs in subclasses 1, 2, 3, 4, 6, 8, and 10 decreased from the WG to the MG stage in both wheat lines (Figure S3). The comparison between DWG and DMG revealed 40 DAAADCs, of which 4 (including one EAA—Trp) were upregulated and 36 (including the EAAs Met, Val, Lys, and Phe) were downregulated (Figure 6A,F and Table S6). Of the 14 DAAADCs identified between YWG and YMG, 1 (the reduced form of Glu) showed an increase, while 13 (including serine (Ser), Met, alanine (Ala), and homocysteine) showed a decrease from the WG to the MG stage (Figure 6B,F and Table S6). These results indicate that the relative contents of DAAADCs significantly differed between the WG and MG stages in the two YHW lines, and that these differences were greater in D than in Y. The number of DAAADCs between DWG and YWG was 26, of which 11 (including Arg and Asn) were upregulated and 15 (all AA derivatives) were downregulated (Figure 6C,F and Table S6). Of the 27 DAAADCs identified between DMG and YMG, 25 (including Lys, Pro, homomethionine, ornithine, His, Thr, Arg, Ser, homoserine, and Asn) increased and 2 (the EAA Trp and the AA derivative N-acetyl-L-tryptophan) decreased (Figure 6D,F and Table S6). Together, these results demonstrate that the relative levels of the DAAADCs in the Y line were higher than those in line D, and that these differences were especially prominent in the MG stage. Furthermore, the high PGC and TAAC of YMG could be attributed to the increased abundance of these AADCs. Overall, 12 DAAADCs were shared between DWG and DMG, and between YWG and YMG, while 4 were shared between DWG and YWG, and between DMG and YMG (Figure 6E and Table S6). This result indicates that the relative abundances of different AADCs in the grains were influenced by both the level of physiological maturity of the grains and the genotypes. These findings provide a scientific basis to determine the optimal harvest time in wheat production.

### 2.5. Transcriptome Analyses of the Two Wheat Lines at Different Developmental Stages

**Figure 7:**
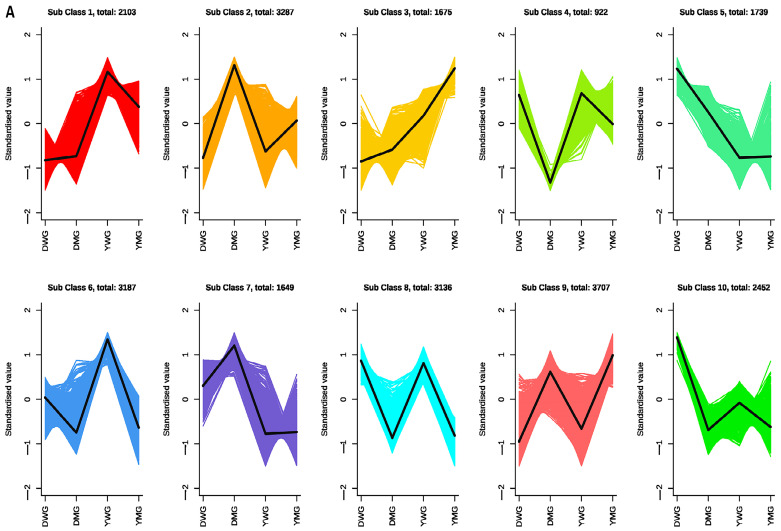

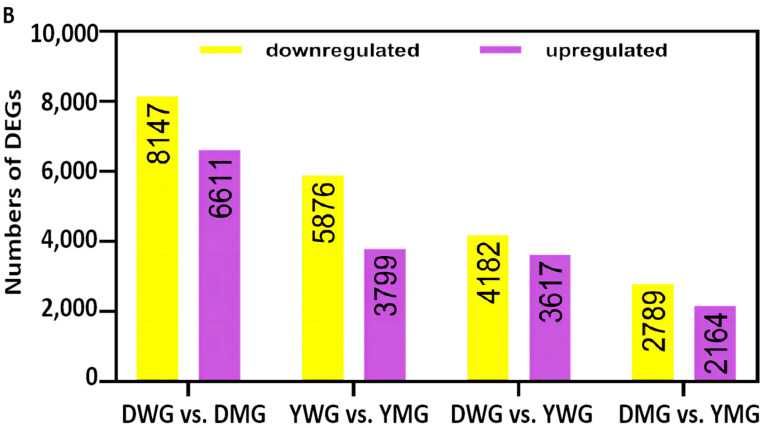
Analysis of the differentially expressed genes (DEGs) among two YHW lines, Dikemai1 (D) and Yunmai 0606 (Y), at two phenological maturation stages, waxy grain (WG) and mature grain (MG): (**A**) Clustered gene expression profiles. The k-means method was used to cluster the DEGs into different subclasses. Groups are presented on the x-axis, while the y-axis represents the standardized fragments per kilobase of transcript per million mapped reads (FPKM) value of each gene within the cluster. (**B**) Number of DEGs between the two sample lines in each comparison group.

To identify potential molecular mechanisms underlying the differences in AAC observed between groups, 12 cDNA libraries (with three biological replicates per group) were constructed from DWG, DMG, YWG, and YMG. In each library, over 93.56% of bases had quality scores ≥30, and the average GC content of the libraries was 58.50%. After filtering for quality, reads were mapped to the reference genome, with the percentage of mapped reads ranging from 82.60% to 95.55% for the different libraries. In total, 16,961 expressed genes and 6896 novel transcripts not annotated in the reference genome were identified (Table S7). A correlation analysis showed significant correlations between the biological replicates within each group (Figure S4A). The PCA demonstrated a clear separation between DWG, DMG, YWG, and YMG, and 43.76% of the total variance could be explained (Figure S4B). Overall, the RNA sequencing (RNA-Seq) data were of high quality and reliability and could be used for further analysis. To identify differentially expressed genes (DEGs) between grains with different levels of physiological maturation, gene expression profiles of WG and MG were combined, and the different groups were compared in a pairwise manner. The k-means method was applied to categorize all 28,257 genes into 10 distinct clusters, indicating that a large number of genes were differentially expressed among different groups (Figure 7A). Using a |log2FC (fold change)| of ≥1 and a false discovery rate (FDR) value of <0.05 as the threshold for the differential expression analysis, a total of 14,758 DEGs (6611 upregulated and 8147 downregulated) were identified between DWG and DMG, while 9675 DEGs (3799 upregulated and 5876 downregulated) were identified between YWG and YMG. On comparing D and Y, 3617 genes were upregulated and 4182 downregulated in the WG stage, while 2164 were upregulated and 2789 downregulated in the MG stage (Figure 7B). 

**Figure 8:**
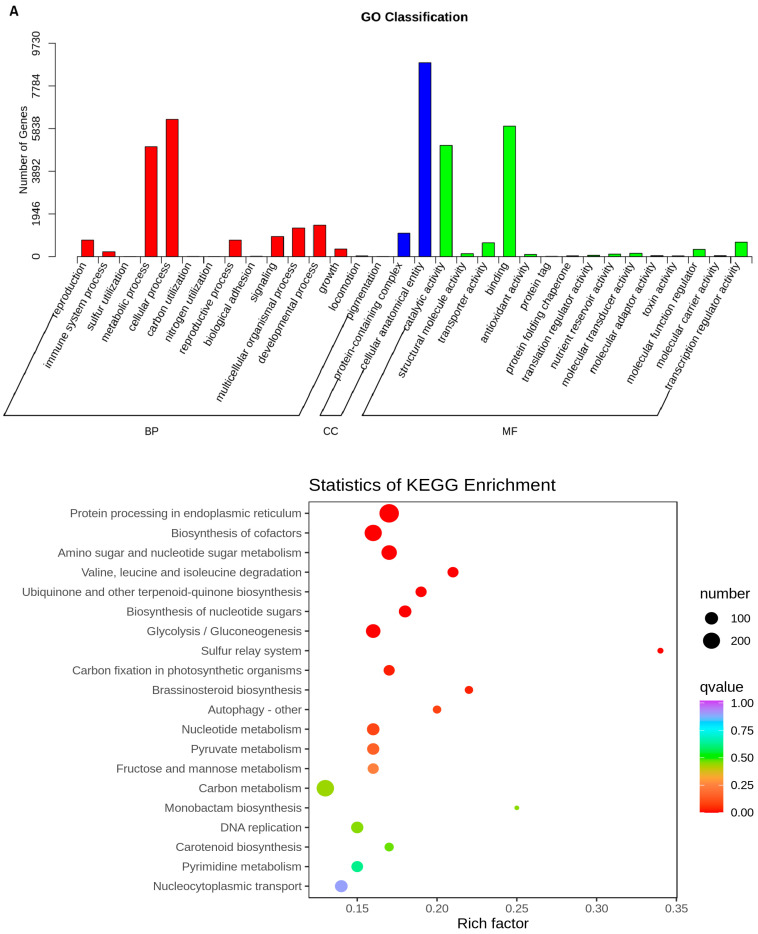

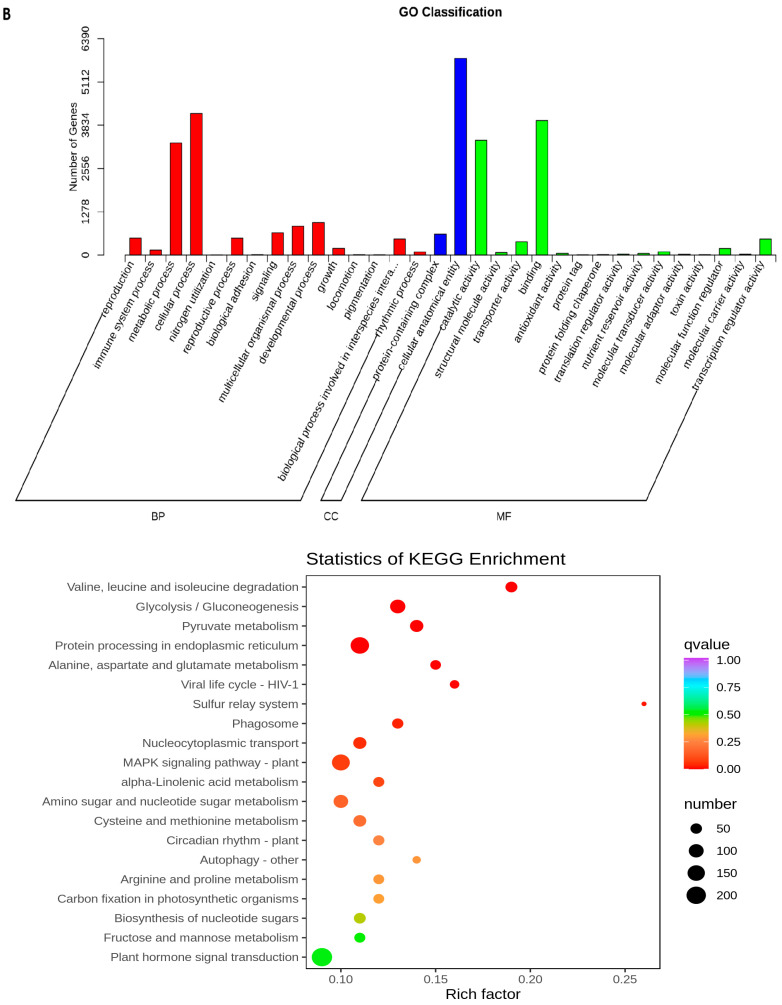

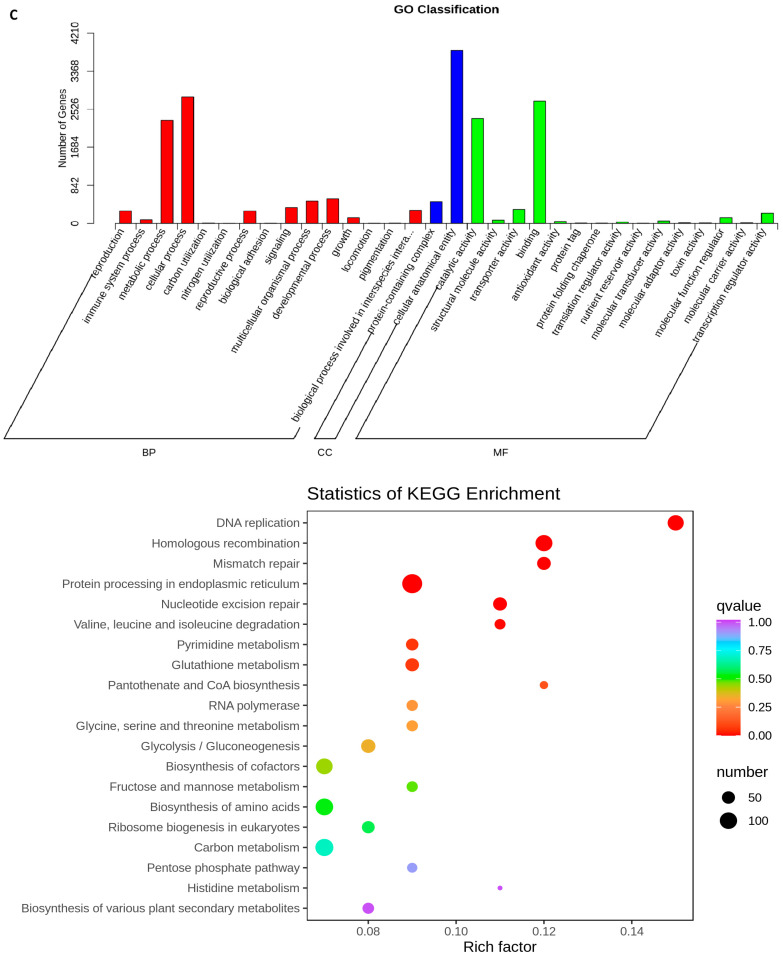

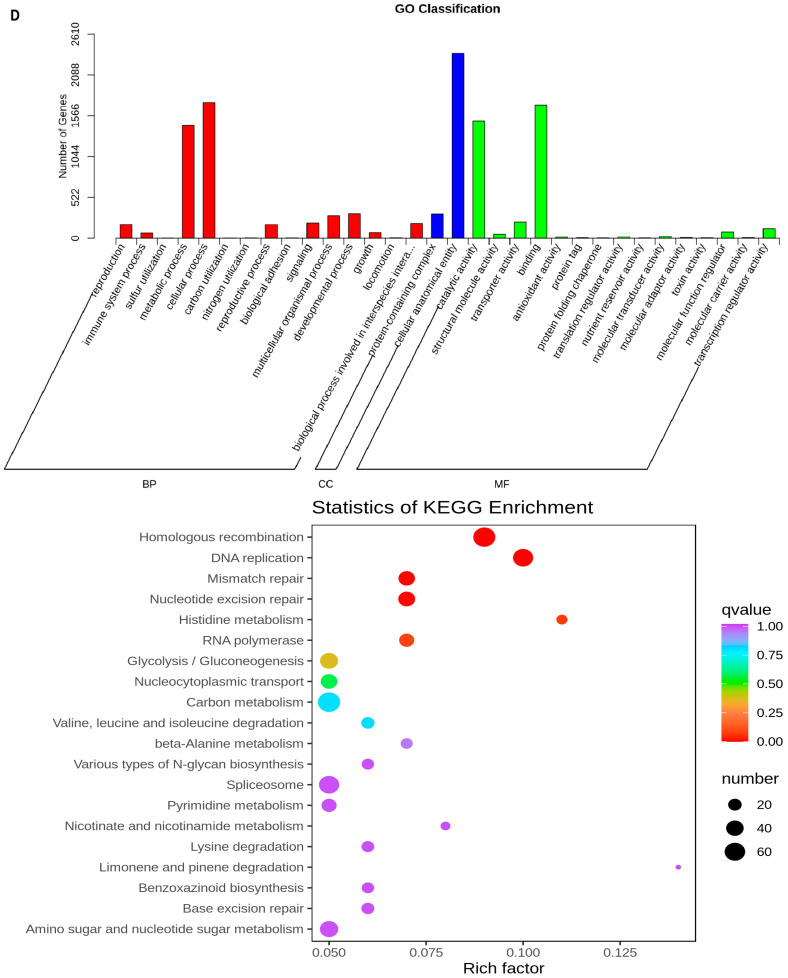
Gene Ontology (GO) classification and Kyoto Encyclopedia of Genes and Genomes (KEGG) pathway enrichment analysis of differentially expressed genes (DEGs) among Dikemai1 (D) and Yunmai 0606 (Y) at two phenological maturation stages, waxy grain (WG) and mature grain (MG): Gene Ontology (GO) classification and the top 20 enriched pathways according to the KEGG analysis, respectively, are shown for the DEGs identified when comparing (**A**) DWG vs. DMG, (**B**) YWG vs. YMG, (**C**) DWG vs. YWG, and (**D**) DMG vs. YMG. BP: biological process; CC: cellular component; MF: molecular function. The size of circles indicates the number of genes. Q-values are indicated by the color scale; *p* < 0.05 was considered statistically significant.

**Figure 9:**
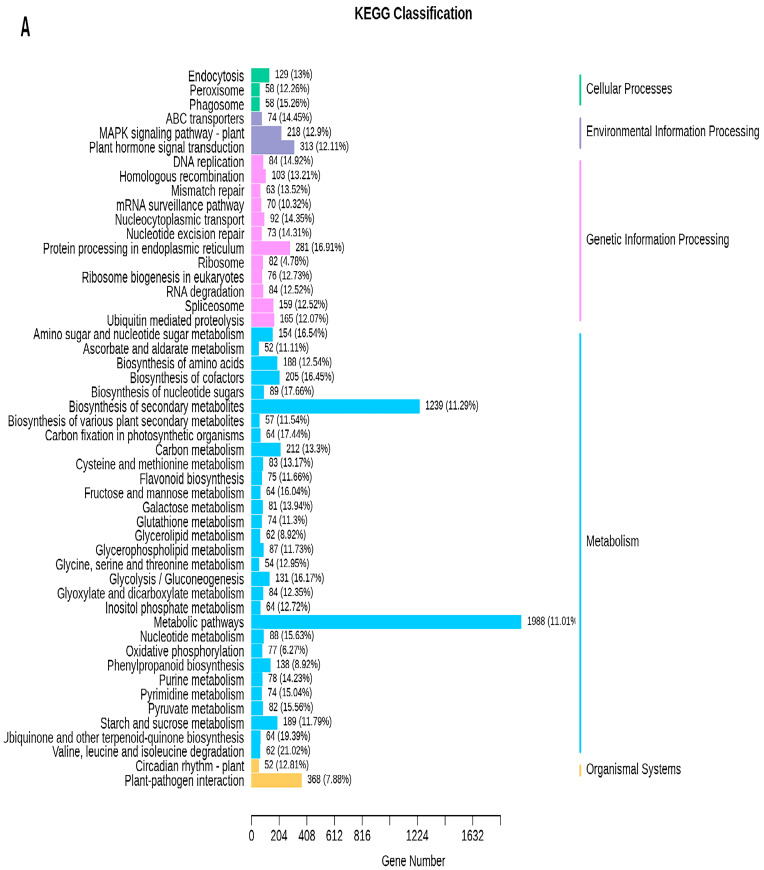

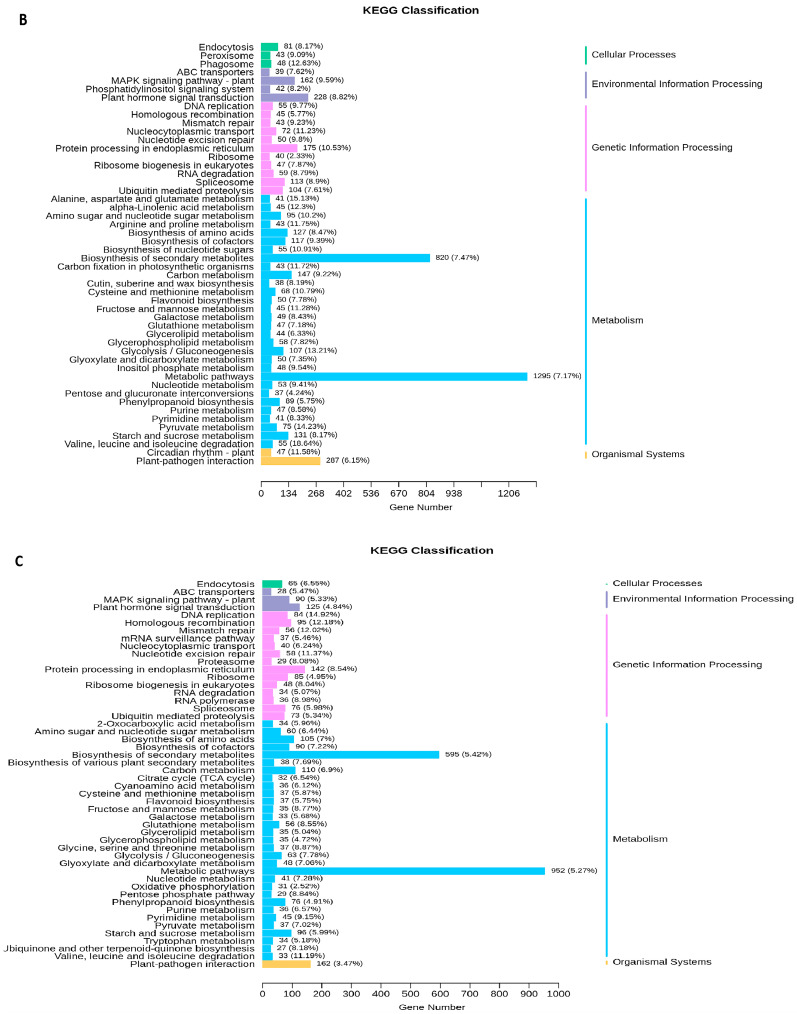

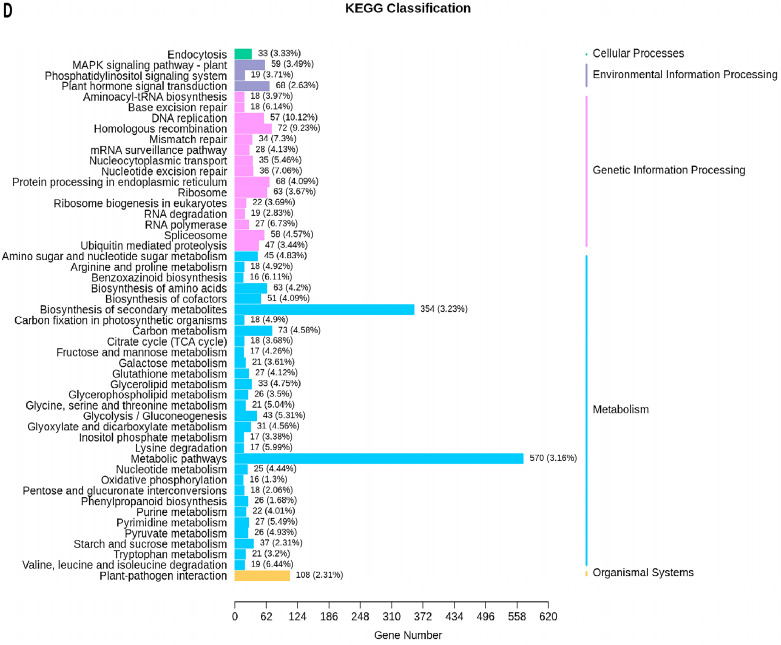
The top 50 pathways involving differentially expressed genes (DEGs) in the different comparison groups, according to the Kyoto Encyclopedia of Genes and Genomes (KEGG). Two wheat lines, Dikemai1 (D) and Yunmai 0606 (Y), at two phenological maturation stages, waxy grain (WG) and mature grain (MG), were compared. The horizontal axis of each graph represents the number of DEGs involved in a specific pathway, and the enriched KEGG pathways are listed on the vertical axis. To the right of each figure, the pathway classifications are indicated. (**A**) DWG vs. DMG, (**B**) YWG vs. YMG, (**C**) DWG vs. YWG, and (**D**) DMG vs. YMG.

To investigate the biological function of DEGs, a Gene Ontology (GO) enrichment analysis was carried out for the different comparison groups. The results showed that DEGs were divided into 41 functional groups (Table S8). Within biological process (BP), cellular process (CP), and metabolic process (MP) were the most prominent terms; anatomical entities were predominantly enriched in cellular component (CC), and binding and catalytic activity were the most represented terms in molecular function (MF) (Figure 8A–D). The metabolic pathways in which DEGs are involved were further identified using KEGG analysis. A total of 4548 (DWG vs. DMG), 3046 (YWG vs. YMG), 2207 (DWG vs. YWG), and 1386 (DMG vs. YMG) DEGs (Table S9) were annotated within 141, 141, 139, and 134 KEGG pathways, of which 34, 32, 28, and 28, respectively, were significantly enriched (*p* < 0.05) (Table S10). Notably, several of the significantly enriched pathways in the different comparison groups directly correlated with the results of the AA analysis: Val, Leu, and Ile degradation (ko00280), Ala, Asp, and Glu metabolism (ko00250), and AA biosynthesis (ko01230) (Figure 8A–D and Table S10). In addition, the enrichment pathways from all four comparison groups can be further divided into five categories: cellular process (CP), environmental information processing (EIP), genetic information processing (GIP), metabolism (M), and organismal systems (OS). Among these categories, M contained the most pathways: the metabolic pathway (ko01100), biosynthesis of secondary metabolites (ko01110), biosynthesis of AAs (ko01230), carbon metabolism (ko01200), biosynthesis of cofactors (ko01240), and starch and sucrose metabolism (ko00500) pathways. These six pathways were the most abundant in the four comparison groups (Figure 9A−D). 

### 2.6. Differential Expression of AA Biosynthesis and Regulatory Genes between WG and MG

**Figure 10:**
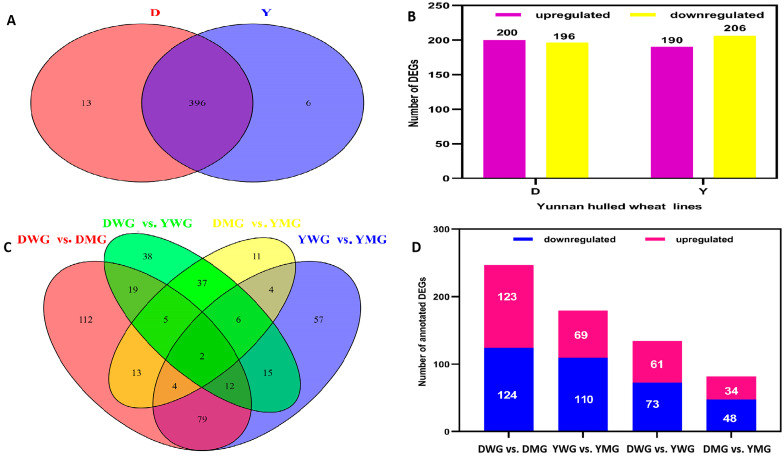
The number of differentially expressed genes (DEGs) in Yunnan hulled wheat lines Dikemai1 (D) and Yunmai 0606 (Y) at two phenological maturation stages, waxy grain (WG) and mature grain (MG): (**A**) Venn diagram showing the total number of DEGs involved in amino acid (AA) biosynthesis in D and Y between the different developmental stages. (**B**) The numbers of up- and downregulated DEGs between the two developmental stages in D and Y. (**C**) The numbers of annotated DEGs involved in AA biosynthesis between the four comparison groups. (**D**) The numbers of up- and downregulated annotated DEGs identified in pairwise comparisons between the four groups.

The KEGG pathway and GO analyses demonstrated that many of the DEGs encode key enzymes and transcription factors involved in AA biosynthesis pathways. These results corroborate the differences in the accumulation of AADCs during the physiological maturation of YHW grains observed in this study. A total of 415 DEGs related to the DAAADCs were detected in D (409) and Y (402) (Table S11), and 396 (more than 95%) of the DEGs overlapped between the two groups (Figure 10A). In D, there was no major difference in the number of upregulated (200) and downregulated (196) DEGs, while in Y, the number of downregulated (206) DEGs was slightly higher than that the number of upregulated DEGs (190) (Figure 10B). Among the DEGs, 235 exhibited the same trend between the WG and MG of both genotypes; of these, 165 were upregulated and 170 were downregulated (Table S12). The Venn diagram shows that two differentially expressed genes are shared in four comparison groups (Figure 10C). The number of downregulated DEGs in YMG was higher than that of upregulated DEGs, and the number of DEGs was significantly higher within the same lines between different physiological stages than between different lines at the same stage (Figure 10D). The expression pattern of up- and downregulated genes showed a trend similar to that of the relative abundance of AADCs in grains between stages of physiological maturity (from WG to MG). In DWG vs. DMG, specifically, 247 DEGs were identified, with an almost equal number of upregulated (123) and downregulated (124) genes. Of the DEGs, 210 were structural genes: 201 encoding structural proteins, 2 encoding glycosyltransferases, and 7 encoding transcription factors (Table S11). The extent to which DEGs between DWG and DMG were up- or downregulated was similar (Figure S5A). The expression pattern observed in Y differed from that in D. Between YWG and YMG, 179 DEGs were identified, and the number of downregulated genes (110) was 1.59 times higher than that of upregulated genes (69). Of the DEGs, 155 were structural genes: 148 encoding structural proteins, 2 encoding glycosyltransferases, and 5 encoding transcription factors (Table S11). The expression levels of genes that were upregulated in YMG were notably lower than those of those in YWG (Figure S5B). Overall, the relative abundance of AADCs in WG was higher than that in MG. Between DWG and YWG, 134 DEGs were found, and the number of upregulated genes (61) was 0.84 times that of downregulated genes (73). The DEGs included a total of 100 structural genes, mainly encoding structural proteins (Table S11). In DMG vs. YMG, 82 DEGs were found, and the number of downregulated genes (48) was 1.41 times that of upregulated genes (34). These DEGs included 58 structural genes, mainly encoding structural proteins (Table S11). Although the number of upregulated DEGs in D was higher than that in Y during the WG stage, the expression levels of the DEGs were relatively similar. Furthermore, the relative abundance of AADCs in DWG was higher than that in YWG (Figure S5C). In the MG stage, both the number of upregulated DEGs and their expression levels were higher in D than in Y (Figure S5D). However, in this stage, the relative abundance of AADCs was higher in Y than in D. 

### 2.7. Confirmation of RNA-Seq Data Using Quantitative Real-Time Polymerase Chain Reaction (qRT-PCR)

**Figure 11:**
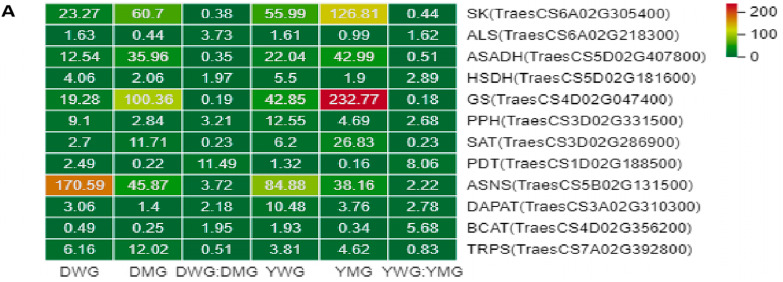

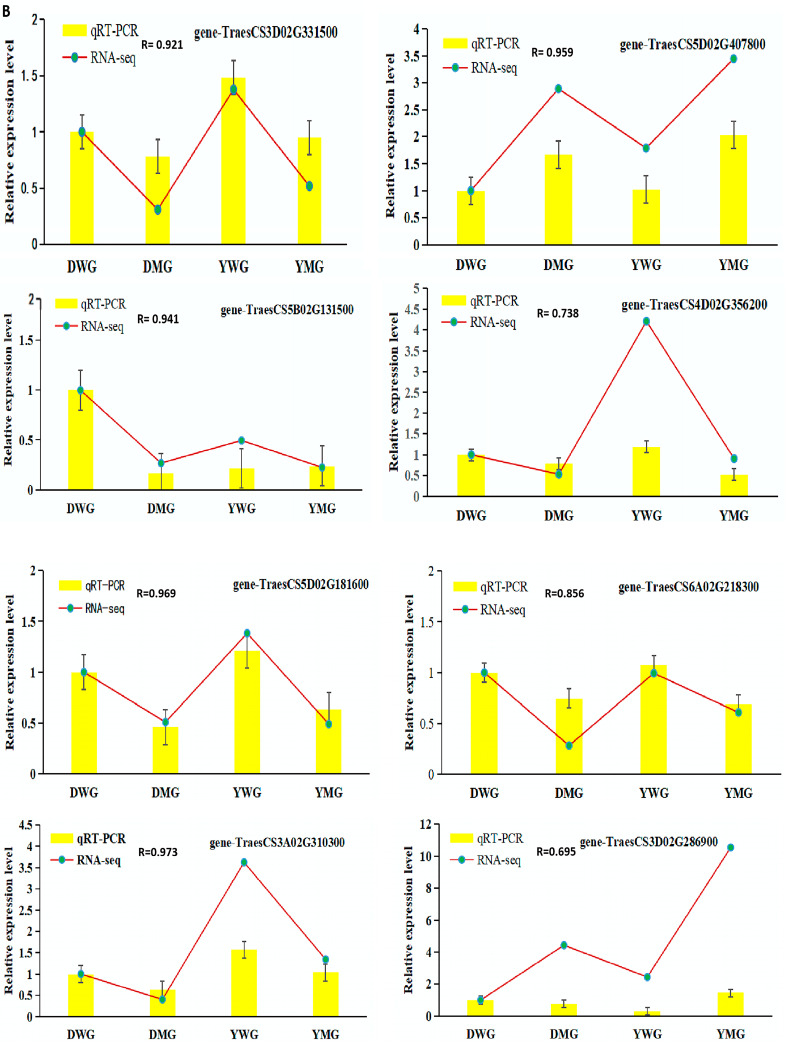

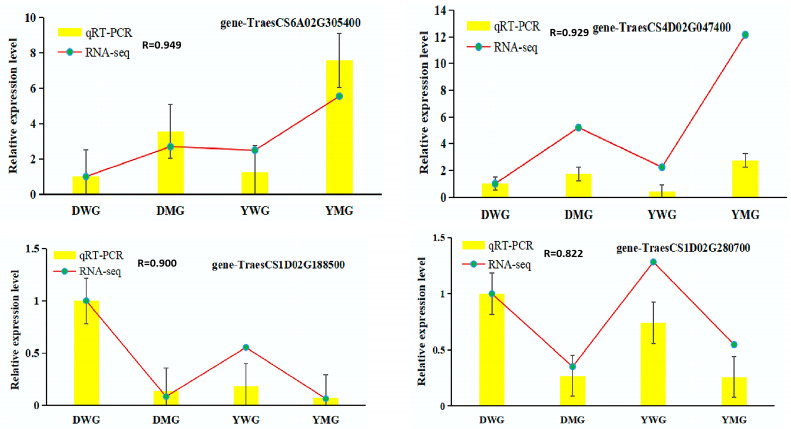
Differentially expressed genes (DEGs) related to amino acid biosynthesis were identified in the RNA sequencing (RNA-Seq) data, and quantitative real-time polymerase chain reaction (qRT-PCR) was used to verify the expression of these genes in two wheat genotypes, Dikemai1 (D) and Yunmai 0606 (Y), at two phenological maturation stages, waxy grain (WG) and mature grain (MG). (**A**) The heatmap shows the fragments per kilobase of transcript per million mapped reads (FPKM) and gene expression differences of DEGs in each group. The color scale shows the relative expression level of DEGs, with red indicating a high level and green indicating a low level. (**B**) The relative expression levels detected by qRT-PCR using the 2^−ΔΔCT^ method are shown by the yellow bars, while the line graph represents the FPKM ratio, indicating transcript abundance in the RNA-Seq data. Values are reported relative to the average gene expression levels in the DWG samples. R is the Pearson’s correlation coefficient between the qRT-PCR and RNA-Seq data. The error bars represent standard errors.

To assess the quality of the RNA-Seq data and confirm differential expression of genes, the expression levels of 12 genes related to AA biosynthesis in the four groups were measured using qRT-PCR. These genes included *TraesCS3D02G331500* encoding enolase (PPH), *TraesCS5B02G131500* encoding asparagine synthase (ASNS), *TraesCS5D02G181600* encoding homoserine dehydrogenase (HSDH), *TraesCS5D02G407800* encoding aspartate-semialdehyde dehydrogenase (ASADH), *TraesCS4D02G356200* encoding branched-chain amino acid aminotransferase (BCAT), *TraesCS6A02G218300* encoding acetolactate synthase (ALS), *TraesCS3A02G310300* encoding diaminopimelate aminotransferase (DAPAT), *TraesCS6A02G305400* encoding shikimate kinase (SK), *TraesCS1D02G188500* encoding prephenate dehydratase (PDT), *TraesCS7A02G392800* encoding tryptophan synthase (TRPS), *TraesCS3D02G286900* encoding serine O-acetyltransferase (SAT), and *TraesCS4D02G047400* encoding glutamine synthetase (GS). The heatmap display (Figure 11A), the expression levels of these genes showed obvious changes in WG and MG, as shown in DWG vs. DMG and YWG vs. YMG, where the differential multiples of genes ranged from 0.19 to 11.49 and from 0.18 to 8.06, respectively. The expression levels of the five genes encoding SK, ASADH, GS, SAT, and TRPS were significantly upregulated in each comparison group, while the expression levels of the genes encoding the other seven enzymes were downregulated in each comparison group. Among them, the gene *TraesCS4D02G047400* encoding GS had the highest upregulation multiple, while the gene *TraesCS1D02G188500* encoding PDT had the highest downregulation multiple. The changes in the expression levels of these genes were essentially consistent with the changes in the contents of related metabolites regulated by the enzymes encoded by the genes (Figure 4). These genes can be used to further verify the reliability of transcriptional data. The changes in expression of the majority of the selected genes, as detected using qRT-PCR, correlated with the results of the RNA-Seq data analysis conducted between the two maturation stages of each group (Figure 11B), indicating the high reliability of the transcriptome data.

### 2.8. Weighted Gene Co-Expression Network Analysis (WGCNA) of AA Biosynthesis during Physiological Maturity

**Figure 12:**
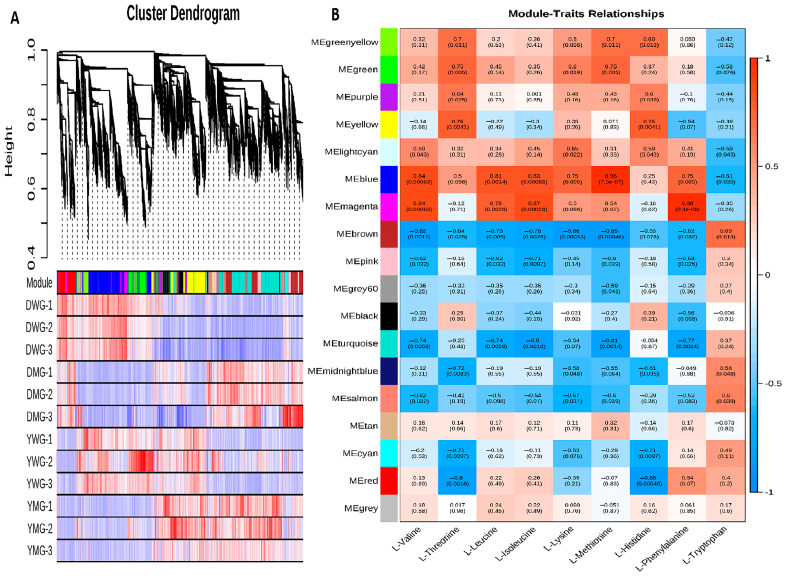
Results of the weighted gene co-expression network analysis (WGCNA): (**A**) A cluster dendrogram grouping genes that exhibited similar expression patterns within samples of the same kind. The red color indicates high expression in samples and modules, while the blue color indicates low expression in samples and modules. Gene modules are shown in different colors. (**B**) Module–trait correlation between differentially expressed genes (grouped in modules) and levels of amino acids (traits). Correlation coefficients (upper row in each block) and *p*-values (in parentheses below the correlation coefficients) for each module–trait pair are shown. Red indicates a strong positive correlation, while blue indicates a strong negative correlation.

WGCNA analysis was carried out on the transcriptomes of grains from the D and Y lines at two physiological maturity stages. The AA metabolome data of grains at each developmental stage (WG and MG) were combined, and the DEGs were divided into 18 modules, each represented by a different color (Figure 12A). Genes enriched within the same module exhibited similar expression patterns between the two developmental stages. The “MEturquoise” module contained the highest number of enriched genes (4431), while the “MEgrey” module contained the lowest number (18). To investigate the gene clusters involved in AA synthesis, a correlation analysis between different gene clusters (modules) and the levels of nine EAAs (traits) was conducted. The results of the module–trait correlation analysis showed that genes in the “MEblue” module showed a significant positive correlation with the levels of six EAAs (Val, Leu, Ile, Phe, Lys, and Met; r > 0.75), while those in the “MEmagenta” module displayed a significant positive correlation with the abundance of Val, Leu, Ile, and Phe (r > 0.78). The level of Lys showed a significant positive correlation with genes in the “MEgreen”, “MElightcyan”, and “MEblue” modules (r > 0.6), while Trp abundance displayed a significant positive correlation with genes in three modules: “MEbrown”, “MEmidnightblue”, and “MEsalmon” (Figure 12B). In addition, the correlation between Trp and the majority of gene modules was the inverse of that of the other eight EAAs with the same modules. The results of the WGCNA analysis demonstrated the complexity of the relationship between AA-biosynthesis-related genes and AAC; the correlation between modules and traits revealed a predominant inverse relationship between the regulation of Trp biosynthesis genes and that of the other eight EAAs.

### 2.9. Identification of the Expression Patterns and Metabolic Pathways of AA Biosynthesis Genes in YHW Lines at Different Stages of Physiological Maturity 

**Figure 13:**
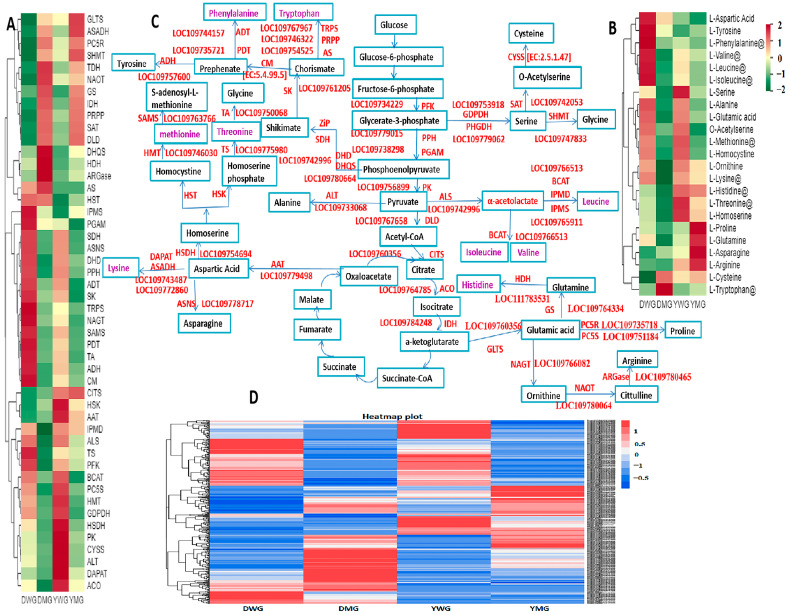
Amino acid (AA) synthesis pathway and gene expression in Dikemai1 (D) and Yunmai 0606 (Y) grains at two phenological maturation stages: waxy grain (WG) and mature grain (MG). (**A**) Heatmap showing expression patterns of major genes related to AA synthesis between WG and MG of D and Y. (**B**) Heatmap showing relative expression levels of the primary AACs between the four groups. Red indicates a high relative level of expression, while green indicates a low relative level of expression. Essential AAs are marked with an “@” symbol. (**C**) Relationships between major genes encoding enzymes involved in the AA synthesis pathway. Essential AAs are highlighted in purple. (**D**) A heatmap of the expression patterns of all differentially expressed genes between WG and MG of D and Y that were shown to be involved in the AA metabolism pathway by the Kyoto Encyclopedia of Genes and Genomes database analysis. Red indicates high gene expression, while blue indicates low gene expression. AAT: aspartate aminotransferase; ACO: aconitate hydratase; ADH: arogenate dehydrogenase; ADT: arogenate dehydratase; ALS: acetolactate synthase; ALT: alanine transaminase; ARGase: arginase; AS: anthranilate synthesis; ASADH: aspartate-semialdehyde dehydrogenase; ASNS: asparagine synthase; BCAT: branched-chain amino acid aminotransferase; CITS: citrate synthase; CM: chorismate mutase; CYSS: cysteine synthase; DAPAT: diaminopimelate aminotransferase; DHD: 3-dehydroquinate dehydratase; DHQS: 3-dehydroquinate synthase; DLD: dihydrolipoamide dehydrogenase; GDPDH: glyceraldehyde 3-phosphate dehydrogenase; GLTS: glutamate synthase; GS: glutamine synthetase; HDH: histidinol dehydrogenase; HMT: homocysteine methyltransferase; HSDH: homoserine dehydrogenase; HSK: homoserine kinase; HST: homoserine O-succinyltransferase; IDH: isocitrate dehydrogenase; IPMD: 3-isopropylmalate dehydrogenase; IPMS: 2-isopropylmalate synthase; NAGT: amino acid N-acetyltransferase; NAOT: acetylornithine aminotransferase; PC5R: pyrroline-5-carboxylate reductase; PC5S: pyrroline-5-carboxylate synthase; PDT: prephenate dehydratase; PFK: 6-phosphofructokinase; PGAM: phosphoglycerate mutase; PK: pyruvate kinase; PPH: enolase; PRPP: ribose-phosphate pyrophosphokinase; SAMS: S-adenosylmethionine synthetase; SAT: serine O-acetyltransferase; SDH: shikimate dehydrogenase; SHMT: serine hydroxymethyltransferase; SK: shikimate kinase; TA: threonine aldolase; TDH: threonine dehydratase; TRPS: tryptophan synthase; TS: threonine synthase.

A total of 415 DEGs (Table S11) related to the accumulation of DAAADCs in the WG and MG stages of YHW lines D and Y were detected. Using correlation cluster heatmap analysis, the relationships between 217 different structural genes and key metabolites in the AA biosynthesis metabolic pathway were analyzed. Of these, 48 genes that were strongly related to AA synthesis were identified (Figure S6 and Table S13). Heatmaps of the expression levels of these genes (Figure 13A) and the abundance of key metabolites in the AA biosynthesis pathway in the four groups (Figure 13B) were drawn. By combining existing knowledge of the AA synthesis pathways identified in other crops and the analyses of the transcriptome and metabolome data of YHW WG and MG, an AA biosynthesis pathway responsible for the physiological maturation of YHW grains was proposed (Figure 13C). The gene expression level heatmap revealed distinctive expression patterns among AA-synthesis-related genes across different YHW varieties at different stages of physiological maturity, leading to their categorization into four groups (Figure 13A): The first group comprised glutamate synthase (GLTS), ASADH, pyrroline-5-carboxylate reductase (PC5R), serine hydroxymethyltransferase (SHMT), threonine dehydratase (TDH), acetylornithine aminotransferase (NAOT), GS, isocitrate dehydrogenase (IDH), ribose-phosphate pyrophosphokinase (PRPP), SAT, and dihydrolipoamide dehydrogenase (DLD). The expression levels of these 11 enzymes were lower in the WG than in the MG of both YHW lines. In contrast, the cumulative expression levels of 29 enzymes in the second group were higher in the WG than in the MG in D and Y. This group contained the following enzymes: 2-isopropylmalate synthase (IPMS), phosphoglycerate mutase (PGAM), shikimate dehydrogenase (SDH), ASNS, 3-dehydroquinate dehydratase (DHD), PPH, arogenate dehydratase (ADT), SK, TRPS, amino-acid N-acetyltransferase (NAGT), PDT, S-adenosylmethionine synthetase (SAMS), threonine aldolase (TA), arogenate dehydrogenase (ADH), and chorismate mutase (CM), all of which exhibited higher expression in DWG than in the other groups. It also contained 14 enzymes that showed elevated expression levels in the YWG group compared to the other groups: 3-isopropylmalate dehydrogenase (IPMD), ALS, threonine synthase (TS), 6-phosphofructokinase (PFK), BCAT, pyrroline-5-carboxylate synthase (PC5S), homocysteine methyltransferase (HMT), glyceraldehyde 3-phosphate dehydrogenase (GDPDH), HSDH, pyruvate kinase (PK), cysteine synthase (CYSS), alanine transaminase (ALT), DAPAT, and aconitate hydratase (ACO). The third group contained five enzymes that were highly expressed in DMG: 3-dehydroquinate synthase (DHQS), histidinol dehydrogenase (HDH), and arginase (ARGase), as well as anthranilate synthase (AS) and homoserine O-succinyltransferase (HST), which were highly expressed in DWG and DMG. The fourth group consisted of three enzymes: citrate synthase (CITS), homoserine kinase (HSK), and aspartate aminotransferase (AAT). Among these, CITS was highly expressed in YMG, while HSK and AAT showed the highest expression levels in YWG. The contents of the different AAs in the grains of different lines of YHW at different maturation stages were directly affected by differences in gene expression levels. The AA heatmap shows that the accumulation levels of different AA species varied between the WG and MG of different lines of YHW; with the exception of six AAs (Trp, Cys, Pro, Arg, Gln, and Asn), the accumulation levels of other AAs in the WG stage were higher than that of those in the MG stage (Figure 13B). This abundance reflects the complexity of the biosynthesis and regulation of AAs. The proposed AA biosynthesis pathway offers novel insights into the intricate interplay between AA metabolites and the expression of genes within the grains of YHW during physiological maturation, providing a foundational framework for investigating AA biosynthesis in YHW or related species (Figure 13C). The expression levels of 415 DEGs involved in the AA biosynthesis pathway within the grains of YHW during physiological maturation were subjected to cluster analysis and visualized using a heatmap (Figure 13D).

### 2.10. Correlation Analysis of EAAs and Transcriptome of YHW Grains at Different Levels of Physiological Maturity

**Figure 14:**
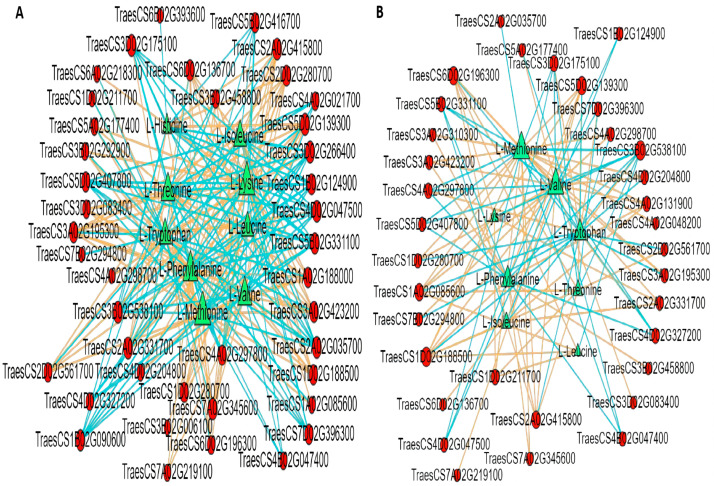

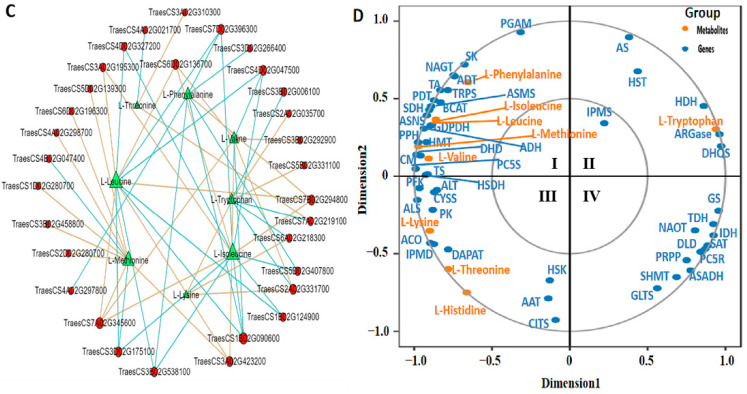
Connection network and canonical correlation analysis (CCA) of essential amino acids (EAAs) and AA-synthesis-related genes during the physiological maturation of Yunnan hulled wheat grains, from the waxy grain (WG) stage to the mature grain (MG) stage. Two lineages, Dikemai1 (D) and Yunmai 0606 (Y), were investigated. The figures illustrate the connection networks between structural genes involved in AA biosynthesis and EAAs of (**A**) DWG vs. DMG, (**B**) YWG vs. YMG, and (**C**) WG vs. MG. The red circle represents differentially expressed genes, while the green triangle represents differentially accumulated metabolites. Solid lines represent a positive correlation, while dashed lines represent a negative correlation (correlation threshold: |r| ≥ 0.80 and *p* < 0.05). Circles represent the primary differentially expressed genes, and triangles represent EAAs. (**D**) CCA of EAAs and genes responsible for their synthesis gene products can be found in the main text.

EAAs greatly impact the nutritional quality of wheat. To deepen our understanding of the regulatory network and key structural genes involved in AA biosynthesis in YHW grains during physiological maturation, Pearson’s correlation analysis was conducted to investigate the relationships between the levels of nine EAAs and 48 structural genes in the WG and MG stages of D and Y. A high-level correlation network diagram was generated using the Metware Cloud Platform (https://cloud.metware.cn, accessed on 28 August 2021), with a correlation coefficient threshold of |r| ≥ 0.80 and *p* < 0.05. The results showed that, between DWG and DMG (Figure 14A), the expression level of *TraesCS2D02G280700* (encoding PPH) was significantly positively correlated with eight other genes. A high-level correlation network diagram was drawn for other EAAs apart from Trp, while the expression of *TraesCS5B02G33100* (encoding DHQS) displayed a significant positive correlation with all EAAs apart from Trp. The expression levels of *TraesCS2A02G415800* (encoding TA), *TraesCS5D02G139300* (encoding ASNS), *TraesCS7A02G345600* (encoding ADH), and *TraesCS2A02G331700* (encoding PFK) showed a significant negative correlation with Trp and a significant positive correlation with the other EAAs, while those of *TraesCS3B02G538100* (encoding PC5R), *TraesCS3D02G266400* (encoding GLTS), *TraesCS4D02G047500* (encoding TDH), *TraesCS2A02G035700* (encoding ARGase), and *TraesCS3D02G175100* (encoding IDH) showed a significant positive correlation with Trp and a significant negative correlation with the other eight EAAs. These results show that the biosynthesis of Trp may be related to the synergistic regulation of multiple genes. Similarly, correlation analysis of the gene expression and AA abundance between YWG and YMG indicated that AA biosynthesis genes may act synergistically or antagonistically. Expression of *TraesCS1D02G188500* (encoding PDT) and *TraesCS3B02G538100* (encoding PC5R) were highly correlated with eight EAAs apart from His, with the former showing a significant negative correlation with Trp and a significant positive correlation with the other EAAs, while the latter exhibited the reverse pattern (Figure 14B). The correlation analyses between the WG and MG stages of both groups showed a strong correlation between gene expression and the abundance of eight EAAs, excluding His. Among these genes, the expression of both *TracesCS7A02G345600* (encoding ADH) and *TracesCS1B02G090600* (encoding NAOT) was correlated with Val, Leu, Ile, and Met, with the former showing a significant positive correlation with these four AAs, while the latter showcased the opposite trend. The abundance of Lys and Trp was also correlated with *TraesCS3B02G006100* (encoding IPMD) and *TraesCS6A02G218300* (encoding ALS), while *TraesCS2A02G331700* (encoding PFK) displayed a significant positive correlation with Lys and a significant negative correlation with Trp (Figure 14C). To further identify the genes encoding key enzymes involved in AA biosynthesis during the maturation of YHW grains, canonical correlation analysis (CCA) was used to analyze the relationship between the nine EAAs and biosynthetic pathway genes (Figure 14D). Dimensionality reduction achieved through CCA indicated that the nine EAAs were distributed across three quadrants and were typically associated with the expression of 12 genes. In the first quadrant, Phe, Leu, Ile, Val, and Met were typically associated with the expression of ADT, GDPDH, ADH, ASNS, PPH, HMT, DHD, and CM. In the second quadrant, Trp was associated with the expression of ARGase and DHQS. In the third quadrant, Lys, Thr, and His were predominantly associated with ACO and IPMD expression. This analysis underscores that key enzyme genes play a constructive role in AA biosynthesis during the physiological maturation of YHW grains. The intricate nature of AA biosynthesis involves a multitude of intricate reaction steps, each demanding precision. AAs are synthesized through the glycolysis pathway (EMP), tricarboxylic acid (TCA) cycle, and pentose phosphate pathway (PPP). Because Lys was the first limiting AA, and Trp showed the highest abundance in the DMG, these AAs could be the focus of research aiming to improve the nutritional quality of wheat. Through the analysis of the high-level correlation network between gene expression levels and those of EAAs, *PPH*, *ADH*, *IPMD*, *DHQS*, and *ARGase* were identified as upstream genes in AA biosynthesis pathways, directly participating in AA synthesis. These genes emerge as pivotal players in AA biosynthesis during the physiological maturation of YHW grains.

## 3. Discussion

Bread wheat grains are used in the development of various health products because of their high nutrient contents. YHW is a hexaploid wheat species, which is considered to be something of value for wheat breeding owing to its good quality and high protein content [39]. However, the understanding of AA composition and the molecular mechanisms involved in AA biosynthesis in YHW grains at the physiological maturity stage is still unclear. This study used two YHW lines, Y (stable high GPC) and D (stable low GPC), revealing their AA profiles and investigating their AA synthesis mechanisms in YHW grains at the stage of physiological maturity by integrating transcriptomic and metabolite analyses. The purpose of this research was to screen for excellent germplasm of YHW from Yunnan, China, with good AA profiles, and to analyze the dynamic changes of AAs in the grains during the physiological maturity development stage of the wheat, as well as identifying candidate genes and gene expression networks that control AA contents. The results improved our understanding of AA compositions in YHW grains. AA accumulation patterns and the mechanisms of changes between WG and MG owing to the key characteristic genes regulating amino acid synthesis were identified. Meanwhile, the research also provided a reference for wheat grains at different filling stages and a framework for further elucidating the molecular mechanisms of transcription and metabolism that lead to higher quality during the WG stage than the MG stage.

Proteins play an important role in improving cereal quality, and their contents are considered to be an indicator of cereal nutritional quality in grains. AAs, as the elementary units of proteins, mediate cell protein synthesis, growth, metabolism, and development [40]. In this study, we detected a total of 103 AAs (including derivatives) and 24 conventional AAs (all L-type) in the physiologically mature grains of YHW, including 19 main AAs in mature wheat grains and 9 EAAs that the human body cannot synthesize and are considered to be restricted EAAs in wheat (Figure 4). These results suggest that the YHW grains were rich in AAs. The AAC in the human body can be supplemented by consuming YHW grains, and the AAC in the D- and Y-line grains at the WG stage was remarkably higher than that in the MG stage (Figure 5). In addition, the AAC of D-line MGs was lower than that of Y-line MGs. These results were consistent with the analysis of physiological and biochemical indicators and indicated differences in AA synthesis and metabolic mechanisms between WG and MG grains in the two YHW lines; however, the composition and content of AAs in wheat are affected by the cultivar, pesticides used in wheat protection, nutrition, cultivation environment, and grain development stage, among others [34,35,37,38,41]. In this study, the Glu/Arg content was the highest, whereas that of Cys/Ala was the lowest. Among the EAAs, the contents of Trp, Met, and His were relatively high in the physiologically mature grains of YHW lines. Meanwhile, Met, Trp, and Phe were the top three EAAs in the WG stage of the D line. His, Phe, and Val were the top three EAAs in the MG stage of the D line, while those in the WG and MG stages of the Y line were Met, His, and Trp and His, Thr, and Phe, respectively. The AAC distribution heatmap showed that only Trp content was higher in the MG stage than in the WG stage, while Val, Thr, Leu, Ile, Lys, Met, His, and Phe contents were lower in the MG stage than in the WG stage (Table S5). The study also showed that although the AA composition of the physiologically mature grains of the two YHW lines was the same, there was a clear difference in content (Figure 13). In addition, throughout physiological maturation, the AA contents within grains of different lines of YHW exhibited substantial differences. The differential accumulation of tryptophan metabolites in grains at varying stages of physiological maturation may be the result of different enzyme activities. Similar trends were observed in both the D and Y lines. Further research is required to determine whether these trends remain consistent across different Yunnan wheat genotypes. This study describes, for the first time, the dynamic changes in AAs in grains of different YHW lines during the developmental stages of physiological maturation.

Lys, Thr, Met, and Ile are the most restricted EAAs in wheat [4,22]. Although there are only 20 types of conventional AAs that constitute proteins, of which 9 EAAs are essential for human nutrition and health, other AAs such as homoserine, ornithine, aspartic acid, and proline are important in terms of regulating growth and development, maintaining cells’ osmotic pressure, and removing antioxidants from ROS (reactive oxygen species) [42,43,44]. The Lys, Thr, Met, and Ile contents at the WG stage in the D line were 3.81-, 1.84-, 12.21-, and 1.60-fold higher than those at the MG stage in the D line, respectively. Similarly, the Lys, Thr, Met, and Ile contents at the WG stage of the Y line were 1.16-, 1.38-, 7.62-, and 1.17-fold higher than those at the MG stage, respectively. Overall, the homoserine, ornithine, Asp, and Pro contents in the MG stage were higher than those in the WG stage. Moreover, although the contents of tryptophan and its derivative 5-hydroxytryptophan in the MG stage were higher than those in the WG stage, the contents of serotonin and its derivative (N-feruloserotonin) were higher in the WG stage than in the MG stage. The accumulation of various Trp metabolites during different developmental stages of physiological maturity may result from different enzymes catalyzing the influx of Trp into different metabolic pathways [45]. Therefore, the AA quality in the waxy-stage grains of different YHW lines was significantly better than that in the mature-stage grains. This also revealed and verified, for the first time, the reason for the high quality of wheat harvested during the waxy stage in wheat production from the perspective of the metabolite content level.

Differential accumulated AAs and derivative compounds (DAAADCs) analysis showed more DAAADCs in the D line than in the Y line at the two physiological maturity stages, and DAAADCs in the WG stage were remarkably higher than those in the MG stage of the two YHW lines, including upregulation and downregulation of different AA metabolites (Figure 6). This was consistent with the DEG results (Figure 10 and Figure S5). Furthermore, the heatmap revealed that Ser, Met, Ala, and homocysteine were the only conventional AAs shared by the two YHW lines during the WG and MG stages; there were also certain differences in DAAADCs between the two Yunnan iron-husk wheat lines. These results indicated that the accumulation of various AAs was influenced by variety and grain development stage, implying that the gene network controlling AA accumulation in grains is fundamentally complex and needs to be elucidated. In-depth analysis of the changes in AA composition and content in YHW grains at different developmental stages, as well as molecular regulation, may contribute to the improvement of wheat’s nutritional quality and breeding utilization.

AA biosynthesis is influenced by the dual metabolism of C and N, resulting in complex regulatory pathways [46]. Different types of AA synthesis utilize intermediates of the Embden–Meyer pathway (EMP), pentose phosphate pathway (PPP), and tricarboxylic acid (TCA) cycle as C chains [47]. The increase in the AA composition and content in grains may be due to increased protein degradation [48], reduced glycolysis, metabolic activation of related nitrogen-containing compounds, increased biosynthesis of AAs, and enhanced expression of glycolytic enzymes that increase the synthesis of certain AAs (such as Glu, Thr, and Cys) [49]. In this study, based on metabolome and transcriptome data, the AA biosynthesis pathway of the two physiological maturity and developmental stages of YHW grains was constructed for the first time (Figure 13). A clustering heatmap of the correlation between the DEGs and the main AAs and their derivatives in the AA biosynthesis pathway is shown in Figure S6. We found that, among 48 enzyme-structure-related genes and 23 AAs and their derivatives in AA biosynthesis pathways, most AA contents were correlated with the expression of their corresponding synthase-structure-related genes, except for TRAP and Trp, GLTS and Glu, SAT and O-acetylserine, HDH and His, CYSS and Cys, and ASNS and Asn. We speculate that the degradation and downstream transformation of these metabolites may be enhanced. We also found that the expression levels of most enzyme-structure-related genes (*PFK*, *PPH*, *PGAM*, *PK*, and *ACO*) involved in the EMP, TCA cycle, and PPP decreased. Compared with those during the WG stage, the EMP and TCA cycles of YHW grains during the MG stage were both downregulated, indicating that the decrease in AA content in the grains during the MG stage may be related to the reduced expression of genes related to carbohydrate metabolism. This is because the EMP-TCA cycle is the main energy source, providing a carbon skeleton for the synthesis of basic metabolites required for plant growth and development [50].

There is a lack of research on the dynamic changes in AA content and composition during grain development, as well as on the enzymes involved in AA synthesis and metabolism—especially the activity changes of metabolic enzymes involved in EAA biosynthesis, such as Lys biosynthesis. Integration of metabolomics and transcriptomics is an effective tool for studying the mechanisms of AA biosynthesis. WGCNA is a powerful tool in systems biology that can be used as a key genetic network to identify the biosynthesis of many crop quality components [51]. In this study, based on the two YHW lines at different physiological maturity development stages, DEG expression levels (fpkm) of transcriptome and the metabolome data of nine kinds of EAAs, a WGCNA was performed to explore the gene cluster related to AA synthesis. WGCNA revealed blue and magenta modules that were significantly and positively correlated with the contents of the four EAAs in YHW (Figure 12). The genes of the two modules were expressed specifically in the WG and MG stages, suggesting that these modules are important in the physiological maturity of developing grains. 

Based on the integrative analyses of the major enzyme-encoding DEGs and nine EAAs, we constructed a high-level correlation network diagram (Figure 14). In line D of YHW, the expression of PPH (encoded by *TraesCS2D02G280700*), ADH (encoded by *TraesCS7A02G345600*), ASNS (encoded by *TraesCS5D02G139300*), TA (encoded by *TraesCS2A02G415800*), PFK (encoded by *TraesCS2A02G331700*), DHQS (encoded by *TraesCS5B02G33100*), PC5R (encoded by *TraesCS3B02G538100*), GLTS (encoded by *TraesCS3D02G266400*), TDH (encoded by *TraesCS4D02G047500*), ARGase (encoded by *TraesCS2A02G035700*), and IDH (encoded by *TraesCS3D02G175100*) was significantly correlated with the contents of nine EAAs, and these may be the candidate hub-enzyme-encoding genes of this line. Similarly, the expression of PDT (encoded by *TraesCS1D02G188500*) and PC5R (encoded by *TraesCS3B02G538100*) was strongly and significantly correlated with the contents of eight EAAs in the Y line, and these were deemed to be the candidate hub-enzyme-encoding genes of this line. Moreover, ADH (encoded by *TracesCS7A02G345600*) and NAOT (encoded by *TracesCS1B02G090600*) were both identified as candidate hub-enzyme-encoding genes of the two YHW lines. Furthermore, the contents of Lys and Trp were strongly and significantly correlated with the expression of IPMD (encoded by *TraesCS3B02G006100*), ALS (encoded by *TraesCS6A02G218300*), and PFK (encoded by *TraesCS2A02G331700*). CCA also identified ADT, GDPDH, ADH, ASNS, PPH, HMT, DHD, CM, ARGase, DHQS, ACO, and IPMD as playing major roles in AA biosynthesis. Overall, the genes *TraesCS2D02G280700, TraesCS7A02G345600, TraesCS3B02G006100, TraesCS5B02G33100,* and *TraesCS2A02G035700* were identified as playing major roles in AA biosynthesis in YHW, which is similar to and different from the enzyme-encoding genes reported to play major roles in other plants [41,46], possibly because of differences in plant species and stages of grain development.

## 4. Materials and Methods

### 4.1. Plant Material and Samples

Fifty YHW lines, maintained at Yunnan Agricultural University, were planted in a randomized complete block design across two locations: Baoshan, Yunnan, China (in 2019) and Xundian, Kunming, Yunnan, China (in 2020). After reaching full maturity, samples were harvested in batches and subjected to near-infrared analyzer analysis (Foss-NIRS DA1650, Copenhagen, Denmark) to determine their GPC. Two YHW lines, Y and D (the origin and source of seeds of YHW lines Yunmai and Dikemail from Lancang and Lincang of Yunnan Province, China, respectively), with stable differences in GPC in both environments, were selected for further analysis. Y and D were planted in greenhouses in 2021. Three biological replicates were collected from the Xundian Dahejiao Experimental Base of Yunnan Agricultural University (25°24′ N, 102°43′ E). Similar-sized spikes that bloomed on the same day during the flowering period were tagged, and the seeds of the two YHW lines were collected every 7 days from 7 DPA until they reached full maturity. Samples were immediately frozen in liquid nitrogen and stored at −80 °C until further analysis.

### 4.2. Measurement of Grain Characteristics

The TAAC was determined using a colorimetric assay kit (A026-1-1; Nanjing Jiancheng Bioengineering Institute, Nanjing, China), following the manufacturer’s instructions. We measured the absorbance of all samples at 650 nm using UV–visible spectrophotometry (UV-8000S; METASH; Shanghai Yuanxi Instrument Co., Ltd., Shanghai, China). Grain morphological parameters, including grain length, width, fresh weight, dry weight, and total weight, were evaluated. The weight (g) per 1000 kernels was measured using an intelligent seed-testing instrument (TPKZ-2; Zhejiang Topu Yunnong Technology Co., Ltd., Zhejiang, China). Statistical significance was set at *p* < 0.05 for multiple-comparisons analysis of variance (ANOVA).

### 4.3. Metabolite Extraction and Profiling

Metabolites were extracted and analyzed by Metware Biotechnology Co., Ltd. (Wuhan, China). Three biological replicates of each of the two YHW lines were collected at 42 and 53 DPA, respectively, and subsequently freeze-dried using a vacuum lyophilizer. Extractions were performed as previously described [52,53]. Briefly, the fine freeze-dried powder from each grain sample (a total of 100 mg in weight) was subjected to extraction using 1.0 mL of 70% (*v*/*v*) aqueous methanol solution, vortexed for 30 s every 30 min for 6 times in total, and then the sample was placed in a refrigerator at 4 °C overnight. After centrifugation for 10 min at 16,260× *g*, the extracts were collected and filtered. The AADCs were analyzed using ultrahigh-performance liquid chromatography (UHPLC, SHIMADZU Nexera X2; https://www.shimadzu.com.cn/, accessed on 28 August 2021) and coupled with electrospray ionization (ESI) tandem mass spectrometry (MS/MS, Applied Biosystems 4500 Q TRAP; https://www.thermofisher.cn/cn/zh/home/brands/applied-biosystems.html, accessed on 28 August 2021). Analyses were performed as previously described [54]. The analytical conditions were as follows: The mobile phase consisted of solvent A (pure water with 0.1% formic acid) and solvent B (acetonitrile with 0.1% formic acid). Sample measurements were performed with a gradient program that employed the starting conditions of 95% A, 5% B. Within 9 min, a linear gradient to 5% A, 95% B was programmed, and a composition of 5% A, 95% B was kept for 1 min. Subsequently, a composition of 95% A, 5.0% B was adjusted within 1.1 min and kept for 2.9 min. The flow velocity was set to 0.35 mL per minute; the injection volume was 4 μL. Then, the extracts were analyzed with an electrospray ionization (ESI) triple-quadrupole linear ion trap (QTRAP)–MS system, and metabolites were qualified using the self-built Metware database (MWDB; http://en.metware.cn/list/27.html, accessed on 28 August 2021). Quantification of AAs and derivatives was performed using multiple reaction monitoring (MRM) with a triple-quadrupole MS [45,52,53,54].

The unsupervised PCA and OPLS-DA were conducted on the UHPLC-MS/MS data using AB Sciex1.6.3 software. HCA of AAs and their derivatives was performed using R software 3.24.3 (www.r-project.org, accessed on 28 August 2021). Differences in accumulated metabolites (DAMs) were considered to be significant at a threshold of |log2FC| ≥ 1 and variable importance in projection (VIP) ≥ 1. We annotated the different metabolites using the KEGG pathway database (http://www.kegg.jp/kegg/pathway.html, accessed on 28 August 2021). The web-based server Metabolite Set Enrichment Analysis (http://www.msea.ca, accessed on 28 August 2021) was used for pathway enrichment analysis.

### 4.4. Transcriptome Sequencing and Data Analysis

High-quality total RNA was extracted as previously described [8,53] and sequenced by Metware Biotechnologies Co. Ltd. Three biological replicates were conducted for each sample type, resulting in a total of 12 transcriptome libraries. Raw RNA sequencing reads were mapped to the Chinese Spring (CS) wheat genome using HISAT2 [55]. StringTie was used to assemble the aligned reads, and gene expression levels were calculated using the FPKM [56]. DESeq2 [57,58] was used to analyze DEGs between sample groups. The *p*-values obtained using the Benjamini–Hochberg method were corrected for FDR. The threshold for DEGs was |log2FC| ≥ 1 and FDR < 0.05. Functional annotation and pathway analyses of DEGs were performed based on seven databases: GO (Gene Ontology), KO (KEGG Orthologdatabase), KOG/COG (Clusters of Orthologous Groups of proteins), Nr (NCBI non-redundant protein sequences), Tr EMBL (Translated EMBL), Pfam (Protein family), and Swiss-Prot (a manually annotated and reviewed protein sequence database) [26].

### 4.5. RT-qPCR

The reliability of the transcriptome sequencing results was verified using RT-qPCR. Total RNA was extracted as previously described [8,40], and the concentration and integrity of RNA were measured using a NanoDrop P 2000 spectrophotometer (Thermo Scientific, Wilmington, DE, USA). Reverse transcription was performed using RNA First-strand cDNA Synthesis SuperMix (TransScript) according to the manufacturer’s instructions. qPCRs were performed using the CFX96 Real-Time System (Touch Thermal cycler, Bio-Rad, California, USA) with the following reaction conditions: 1 cycle at 95 °C for 30 s; 38 cycles at 95 °C for 5 s, 60 °C for 30 s, 95 °C for 10 s, and 65 °C for 5 s; and 4 °C until the end of the run. The ATP-dependent 26S proteasomal regulatory subunit (26S) was used as an internal reference gene, and gene-specific primers (Table S14) were designed using Beacon Designer 7.9. Gene targets for validation were selected randomly. The relative expression levels of genes were calculated using the 2^−ΔΔCT^ method [59].

### 4.6. Co-Expression Network Construction

Weighted gene co-expression network analysis (WGCNA) of DEGs and EAACs of D and Y grains at different stages of physiological maturation was performed using the Metware Cloud (https://cloud.metware.cn, accessed on 28 August 2021).

### 4.7. CCA

We performed CCA on metabolomic and transcriptomic data to explore the correlation between AA metabolite levels and the expression of AA-synthesis-related genes. The EAA contents and expression levels of 48 primary genes in the AA synthesis pathway were determined, and CAA was conducted using Cytoscape software (v3.8.0, http://esr.shriweiwlan.cn/Cytoscape/s2.html, accessed on 28 August 2021).

## 5. Conclusions

YHW grains are wheat germplasm rich in AAs. A total of 103 AAs and derivatives were detected in the two YHW lines. All contained 24 CAAs, of which 9 were EAAs. Although the AA composition of each line was similar, there were significant differences in the contents. There were 40 and 16 differential metabolites in the D and Y grains during the WG and MG stages, respectively. In addition, the abundance of AAs in grains decreased with physiological maturation, and the AA content in the WG stage was higher than that in the MG stage. The Trp content in the grains of the two YHW lines during the MG stage was higher than that in the WG stage; the relative content was the highest in the DMG. The contents of the other eight EAAs in the WG stage were higher than those in the MG stage, and the Lys contents were limited. The Met and Thr contents were the highest in the WG stage of the D and Y lines, respectively. Functional enrichment analysis of transcriptome genes showed that 415 DEGs were related to AA synthesis, and the gene expression patterns related to AA accumulation in physiologically mature grains were consistent with the AA contents. Through cluster heatmap analysis of differentially expressed structural genes and metabolite correlation of AA synthesis pathways, 48 genes were compared and identified as being directly involved in AA synthesis of the two YHW lines’ grains during the WG and MG stages. WGCNA and CCA, combined with metabolomic and transcriptome analyses, revealed important information about AA synthesis and differential accumulation in physiologically mature grains of YHW. The results showed that there were differences in hub genes, including *PPH*, *ADH*, *IPMD*, *DHQS*, and *ARGase*, which were upstream of the AA biosynthesis pathway, or directly critical genes for AA synthesis. The different expression patterns of homologous genes also indicated the synergistic effects of multiple genes in AA synthesis. This study provides information on wheat grains at different maturation stages and lays the foundation for further elucidation of the molecular mechanisms of transcription and metabolism that may lead to higher grain quality during the WG stage than the MG stage.

## Figures and Tables

**Table 1:** The total amino acids and derivative compounds identified.

Compounds	Molecular Weight (Da)	Formula	Class
L-Valine	1.17 × 10^2^	C_5_H_11_NO_2_	Essential amino acid
L-Threonine	1.19 × 10^2^	C_4_H_9_NO_3_	Essential amino acid
L-Leucine	1.31 × 10^2^	C_6_H_13_NO_2_	Essential amino acid
L-Isoleucine	1.31 × 10^2^	C_6_H_13_NO_2_	Essential amino acid
L-Lysine	1.46 × 10^2^	C_6_H_14_N_2_O_2_	Essential amino acid
L-Methionine	1.49 × 10^2^	C_5_H_11_NO_2_S	Essential amino acid
L-Histidine	1.55 × 10^2^	C_6_H_9_N_3_O_2_	Essential amino acid
L-Phenylalanine	1.65 × 10^2^	C_9_H_11_NO_2_	Essential amino acid
L-Tryptophan	2.04 × 10^2^	C_11_H_12_N_2_O_2_	Essential amino acid
L-Alanine	8.90 × 10^1^	C_3_H_7_NO_2_	Amino acids
L-Serine	1.05 × 10^2^	C_3_H_7_NO_3_	Amino acids
L-Proline	1.15 × 10^2^	C_5_H_9_NO_2_	Amino acids
L-Homoserine	1.19 × 10^2^	C_4_H_9_NO_3_	Amino acids
L-Cysteine	1.21 × 10^2^	C_3_H_7_NO_2_S	Amino acids
L-Norleucine	1.31 × 10^2^	C_6_H_13_NO_2_	Amino acids
L-Asparagine	1.32 × 10^2^	C_4_H_8_N_2_O_3_	Amino acids
L-Ornithine	1.32 × 10^2^	C_5_H_12_N_2_O_2_	Amino acids
L-Aspartic acid	1.33 × 10^2^	C_4_H_7_NO_4_	Amino acids
L-Glutamine	1.46 × 10^2^	C_5_H_10_N_2_O_3_	Amino acids
L-Glutamic acid	1.47 × 10^2^	C_5_H_9_NO_4_	Amino acids
L-Homomethionine	1.63 × 10^2^	C_6_H_13_NO_2_S	Amino acids
L-Arginine	1.74 × 10^2^	C_6_H_14_N_4_O_2_	Amino acids
L-Tyrosine	1.81 × 10^2^	C_9_H_11_NO_3_	Amino acids
L-Homocystine	2.68 × 10^2^	C_8_H_16_N_2_O_4_S_2_	Amino acids
N-Methylglycine	8.90 × 10^1^	C_3_H_7_NO_2_	Amino acid derivatives
L-Azetidine-2-carboxylic acid	1.01 × 10^2^	C_4_H_7_NO_2_	Amino acid derivatives
Methyl 3-aminopropanoate	1.03 × 10^2^	C_4_H_9_NO_2_	Amino acid derivatives
N,N-Dimethylglycine	1.03 × 10^2^	C_4_H_9_NO_2_	Amino acid derivatives
N-Acetyl-L-glycine	1.17 × 10^2^	C_4_H_7_NO_3_	Amino acid derivatives
N-Methyl-α-aminoisobutyric acid	1.17 × 10^2^	C_5_H_11_NO_2_	Amino acid derivatives
5-Oxoproline	1.29 × 10^2^	C_5_H_7_NO_3_	Amino acid derivatives
5-Oxo-L-proline	1.29 × 10^2^	C_5_H_7_NO_3_	Amino acid derivatives
Cycloleucine	1.29 × 10^2^	C_6_H_11_NO_2_	Amino acid derivatives
N-Acetyl-beta-alanine	1.31 × 10^2^	C_5_H_9_NO_3_	Amino acid derivatives
Trans-4-hydroxy-L-proline	1.31 × 10^2^	C_5_H_9_NO_3_	Amino acid derivatives
S-Methyl-L-cysteine	1.35 × 10^2^	C_4_H_9_NO_2_S	Amino acid derivatives
L-Cyclopentylglycine	1.43 × 10^2^	C_7_H_13_NO_2_	Amino acid derivatives
N-Methyl-trans-4-hydroxy-L-proline	1.45 × 10^2^	C_6_H_11_NO_3_	Amino acid derivatives
L-Threo-3-methylaspartate	1.47 × 10^2^	C_5_H_9_NO_4_	Amino acid derivatives
O-Acetylserine	1.47 × 10^2^	C_5_H_9_NO_4_	Amino acid derivatives
(2S,3R,4S)-4-Hydroxyisoleucine	1.47 × 10^2^	C_6_H_13_NO_3_	Amino acid derivatives
4-Hydoxy-L-isoleucine	1.47 × 10^2^	C_6_H_13_NO_3_	Amino acid derivatives
N-Acetyl-L-threonine	1.61 × 10^2^	C_6_H_11_NO_4_	Amino acid derivatives
N-Methyl-L-glutamate	1.61 × 10^2^	C_6_H_11_NO_4_	Amino acid derivatives
3-Hydroxy-3-methylpentane-1,5-dioic acid	1.62 × 10^2^	C_6_H_10_O_5_	Amino acid derivatives
4-Hydroxy-L-glutamic acid	1.63 × 10^2^	C_5_H_9_NO_5_	Amino acid derivatives
L-Methionine methyl ester	1.63 × 10^2^	C_6_H_13_NO_2_S	Amino acid derivatives
L-Methionine sulfoxide	1.65 × 10^2^	C_5_H_11_NO_3_S	Amino acid derivatives
L-Glycyl-L-proline	1.72 × 10^2^	C_7_H_12_N_2_O_3_	Amino acid derivatives
N-Acetyl-L-leucine	1.73 × 10^2^	C_8_H_15_NO_3_	Amino acid derivatives
N-Alpha-acetyl-L-asparagine	1.74 × 10^2^	C_6_H_10_N_2_O_4_	Amino acids derivatives
N-α-Acetyl-L-ornithine	1.74 × 10^2^	C_7_H_14_N_2_O_3_	Amino acid derivatives
N-Acetyl-L-aspartic acid	1.75 × 10^2^	C_6_H_9_NO_5_	Amino acid derivatives
O-Phospho-L-serine	1.85 × 10^2^	C_3_H_8_NO_6_P	Amino acid derivatives
N-Acetyl-L-glutamine	1.88 × 10^2^	C_7_H_12_N_2_O_4_	Amino acid derivatives
N-Glycyl-L-leucine	1.88 × 10^2^	C_8_H_16_N_2_O_3_	Amino acid derivatives
L-Glycyl-L-isoleucine	1.88 × 10^2^	C_8_H_16_N_2_O_3_	Amino acid derivatives
N6-Acetyl-L-lysine	1.88 × 10^2^	C_8_H_16_N_2_O_3_	Amino acid derivatives
Homoarginine	1.88 × 10^2^	C_7_H_16_N_4_O_2_	Amino acid derivatives
N-Monomethyl-L-arginine	1.88 × 10^2^	C_7_H_16_N_4_O_2_	Amino acid derivatives
Trimethyllysine	1.88 × 10^2^	C_9_H_20_N_2_O_2_	Amino acid derivatives
N-Acetyl-L-glutamic acid	1.89 × 10^2^	C_7_H_11_NO_5_	Amino acid derivatives
N-Acetyl-L-methionine	1.91 × 10^2^	C_7_H_13_NO_3_S	Amino acid derivatives
L-Tyrosine methyl ester	1.95 × 10^2^	C_10_H_13_NO_3_	Amino acid derivatives
3,4-Dihydroxy-L-phenylalanine (L-dopa)	1.97 × 10^2^	C_9_H_11_NO_4_	Amino acid derivatives
NG,NG-Dimethyl-L-arginine	2.02 × 10^2^	C_8_H_18_N_4_O_2_	Amino acid derivatives
N-Acetyl-L-phenylalanine	2.07 × 10^2^	C_11_H_13_NO_3_	Amino acid derivatives
6-Hydroxydopaquinone	2.11 × 10^2^	C_9_H_9_NO_5_	Amino acid derivatives
N-Acetyl-L-arginine	2.16 × 10^2^	C_8_H_16_N_4_O_3_	Amino acid derivatives
5-L-Glutamyl-L-amino acid	2.18 × 10^2^	C_8_H_14_N_2_O_5_	Amino acid derivatives
5-Hydroxy-L-tryptophan	2.20 × 10^2^	C_11_H_12_N_2_O_3_	Amino acid derivatives
Glycylphenylalanine	2.22 × 10^2^	C_11_H_14_N_2_O_3_	Amino acid derivatives
L-Glycyl-L-phenylalanine	2.22 × 10^2^	C_11_H_14_N_2_O_3_	Amino acid derivatives
N-Acetyl-L-tyrosine	2.23 × 10^2^	C_11_H_13_NO_4_	Amino acid derivatives
L-Prolyl-L-leucine	2.28 × 10^2^	C_11_H_20_N_2_O_3_	Amino acid derivatives
L-Valyl-L-leucine	2.30 × 10^2^	C_11_H_22_N_2_O_3_	Amino acid derivatives
L-Lysine-butanoic acid	2.34 × 10^2^	C_10_H_22_N_2_O_4_	Amino acid derivatives
L-Alanyl-L-phenylalanine	2.36 × 10^2^	C_12_H_16_N_2_O_3_	Amino acid derivatives
L-Leucyl-L-leucine	2.44 × 10^2^	C_12_H_24_N_2_O_3_	Amino acid derivatives
N-Acetyl-L-tryptophan	2.46 × 10^2^	C_13_H_14_N_2_O_3_	Amino acid derivatives
L-Isoleucyl-L-aspartate	2.46 × 10^2^	C_10_H_18_N_2_O_5_	Amino acid derivatives
γ-Glutamyl-L-valine	2.46 × 10^2^	C_10_H_18_N_2_O_5_	Amino acid derivatives
L-γ-Glutamyl-L-leucine	2.60 × 10^2^	C_11_H_20_N_2_O_5_	Amino acid derivatives
Glycyl-tryptophan	2.61 × 10^2^	C_13_H_15_N_3_O_3_	Amino acid derivatives
L-Prolyl-L-phenylalanine	2.62 × 10^2^	C_14_H_18_N_2_O_3_	Amino acid derivatives
Phenylacetyl-L-glutamine	2.64 × 10^2^	C_13_H_16_N_2_O_4_	Amino acid derivatives
L-Valyl-L-phenylalanine	2.64 × 10^2^	C_14_H_20_N_2_O_3_	Amino acid derivatives
γ-Glutamylmethionine	2.78 × 10^2^	C_10_H_18_N_2_O_5_S	Amino acid derivatives
L-Aspartyl-L-phenylalanine	2.80 × 10^2^	C_13_H_16_N_2_O_5_	Amino acid derivatives
γ-Glutamylphenylalanine	2.94 × 10^2^	C_14_H_18_N_2_O_5_	Amino acid derivatives
Glutathione reduced form	3.07 × 10^2^	C_10_H_17_N_3_O_6_S	Amino acid derivatives
γ-Glutamyltyrosine	3.10 × 10^2^	C_14_H_18_N_2_O_6_	Amino acid derivatives
L-Phenylalanyl-L-phenylalanine	3.12 × 10^2^	C_18_H_20_N_2_O_3_	Amino acid derivatives
S-(Methyl)glutathione	3.21 × 10^2^	C_11_H_19_N_3_O_6_S	Amino acid derivatives
Tryptophan glutamic acid	3.33 × 10^2^	C_16_H_19_N_3_O_5_	Amino acid derivatives
S-(5′-Adenosy)-L-homocysteine	3.84 × 10^2^	C_14_H_20_N_6_O_5_S	Amino acid derivatives
Oxiglutatione	6.12 × 10^2^	C_20_H_32_N_6_O_12_S_2_	Amino acid derivatives
L-Alanyl-L-alanine	1.60 × 10^2^	C_6_H_12_N_2_O_3_	Oligopeptides
Cyclo(L-Ala-L-Pro)	1.68 × 10^2^	C_8_H_12_N_2_O_2_	Oligopeptides
Cyclo(D-Leu-L-Pro)	2.10 × 10^2^	C_11_H_18_N_2_O_2_	Oligopeptides
Cyclo(D-Phe-L-Pro)	2.44 × 10^2^	C_14_H_16_N_2_O_2_	Oligopeptides
Cyclo(Pro-Phe)	2.44 × 10^2^	C_14_H_16_N_2_O_2_	Oligopeptides
γ-Glu-Cys	2.50 × 10^2^	C_8_H_14_N_2_O_5_S	Oligopeptides

## Data Availability

No data were used for the research described in this article. The datasets presented in this study can be found in online repositories.

## Supplementary Materials

The following supporting information can be downloaded at: https://www.mdpi.com/article/10.3390/ijms241713475/s1.

## Abbreviations

YHW, Yunnan hulled wheat; AA, amino acid, AAs, amino acids; EAAs, essential amino acids; GPC, grain protein content; AAC, amino acid content; EAAC, essential amino acid content; WG, waxy grain; MG, mature grain; DPA, days post-anthesis; TAAC, total amino acids content; AADCs, amino acids and derivative compounds; CAACs, conventional amino acid components; CAAs, conventional amino acids; DAAADCs, differentially accumulated amino acids and derivative compounds; DEGs, differentially expressed genes; FDR, false discovery rate; FPKM, fragments per kilobase of transcript per million mapped reads; BP, biological process; CC, cellular component; MF, molecular function; CP, cellular process; MP, metabolic process; M, metabolism; EIP, environmental information processing; GIP, genetic information processing; OS, organismal systems; PPH, encoding enolase; ASNS, asparagine synthase; HSDH, homoserine dehydrogenase; ASADH, aspartate-semialdehyde dehydrogenase; BCAT, branched-chain amino acid aminotransferase; ALS, acetolactate synthase; DAPAT, diaminopimelate aminotransferase; SK, shikimate kinase; PDT, prephenate dehydratase; TRPS, tryptophan synthase; SAT, serine O-acetyltransferase; GS, glutamine synthetase; PC5R, pyrroline-5-carboxylate reductase; SHMT, serine hydroxymethyltransferase; GLTS, glutamate synthase; TDH, threonine dehydratase; NAOT, acetylornithine aminotransferase; IDH, isocitrate dehydrogenase; PRPP, ribose-phosphate pyrophosphokinase; DLD, dihydrolipoamide dehydrogenase; IPMS, 2-isopropylmalate synthase; PGAM, phosphoglycerate mutase; SDH, shikimate dehydrogenase; ADT, arogenate dehydratase; NAGT, amino acid N-acetyltransferase; SAMS, S-adenosylmethionine synthetase; TA, threonine aldolase; ADH, arogenate dehydrogenase; CM, chorismate mutase; IPMD, 3-isopropylmalate dehydrogenase; DHD, 3-dehydroquinate dehydratase; PC5S, pyrroline-5-carboxylate synthase; TS, threonine synthase; PFK, 6-phosphofructokinase; HMT, homocysteine methyltransferase; GDPDH, glyceraldehyde 3-phosphate dehydrogenase; PK, pyruvate kinase; CYSS, cysteine synthase; ALT, alanine transaminase; ACO, aconitate hydratase; DHQS, 3-dehydroquinate synthase; HDH, histidinol dehydrogenase; CITS, citrate synthase; ARGase, arginase; AS, anthranilate synthase; HST, homoserine O-succinyltransferase; AAT, aspartate aminotransferase; HSK, homoserine kinase; WGCNA, weighted gene co-expression network analysis; CCA, canonical correlation analysis; EMP, glycolysis pathway; TCA, tricarboxylic acid; PPP, pentose phosphate pathway; ROS, reactive oxygen species; DAMs, differences in accumulated metabolites.
