# Supplementary material for: Integrated Metabolome and Transcriptome Analyses Reveal Amino Acid Biosynthesis Mechanisms during the Physiological Maturity of Grains in Yunnan Hulled Wheat (Triticum aestivum ssp. yunnanense King)
Source: Int J Mol Sci. 2023 Aug 30;24(17):13475. doi: 10.3390/ijms241713475 (PMC10487551; doi:10.3390/ijms241713475)
Supplement: Supplementary file 1 [file ijms-24-13475-s001.zip › Figures S1-S6.pdf]

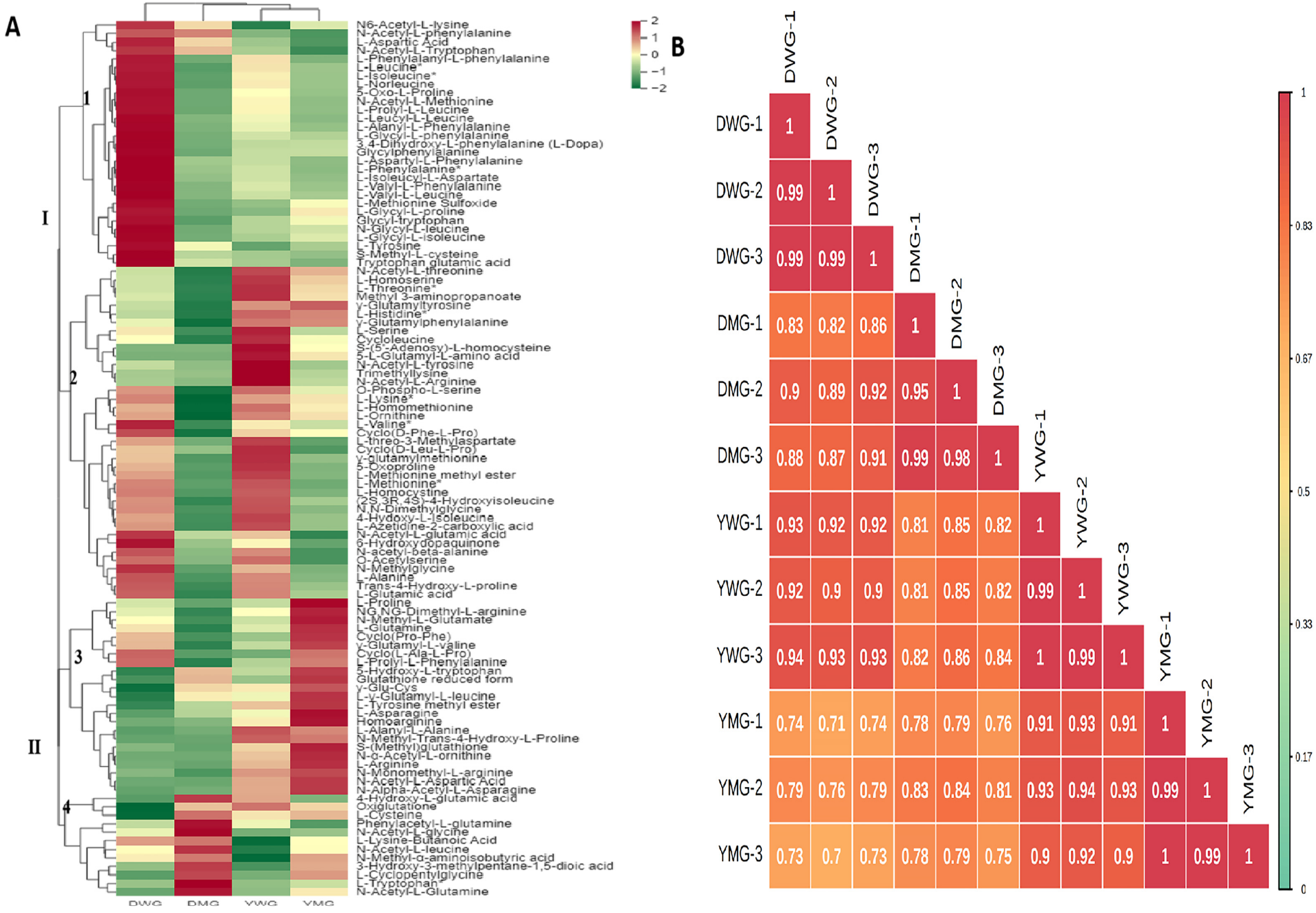

Figure S1. Cluster heatmap and correlation analysis of amino acids and derivative compounds among comparison group.

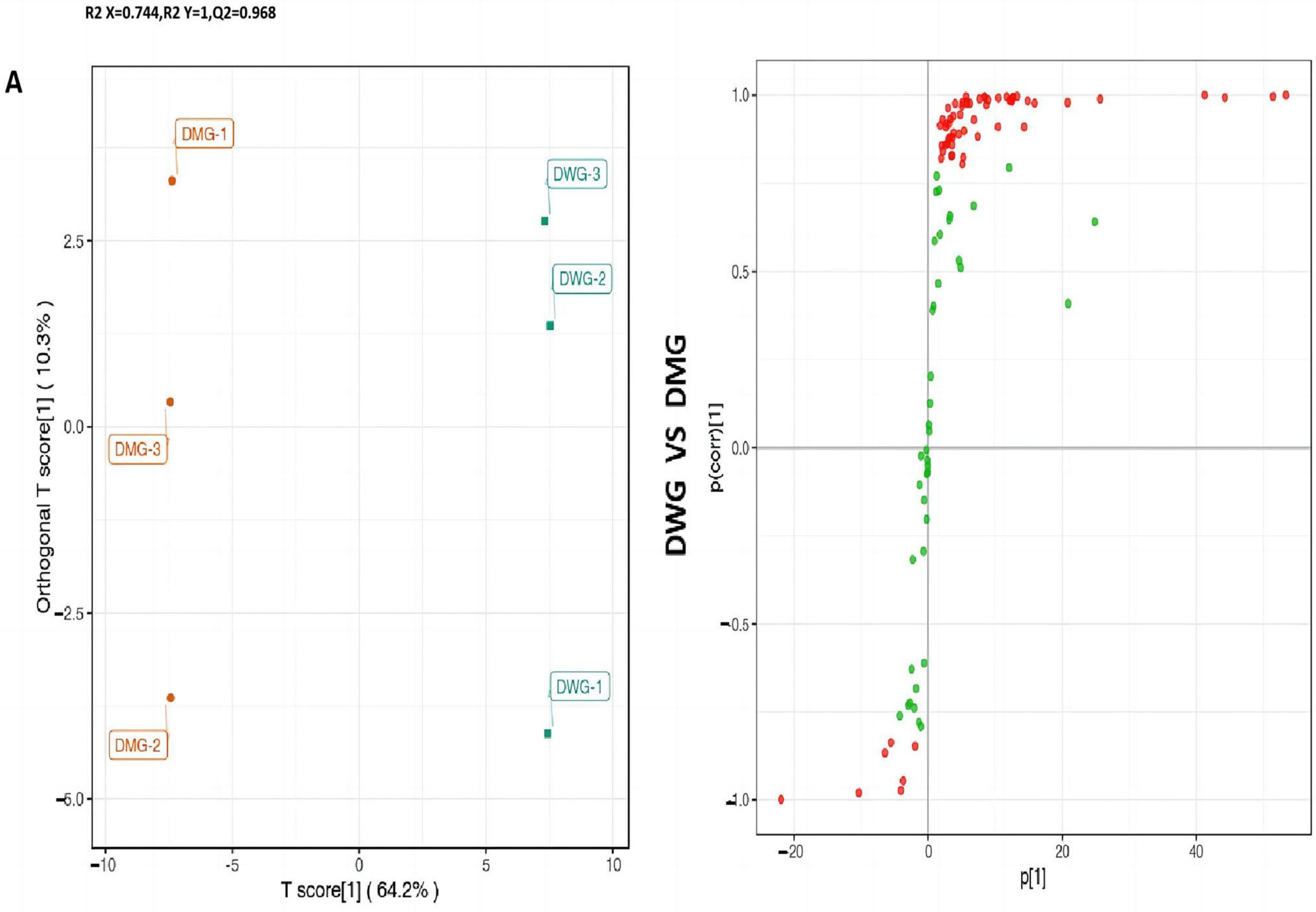
R2 X=0.744,R2 Y=1,Q2=0.968
A
DMG-1
DWG-3
DWG-2
DMG-3
DMG-2
DWG-1
Orthogonal T score[1] (10.3%)
T score[1] (64.2%)
DWG VS DMG
p(corr)[1]
p[1]

R2 X=0.655,R2 Y=1,Q2=0.967

B

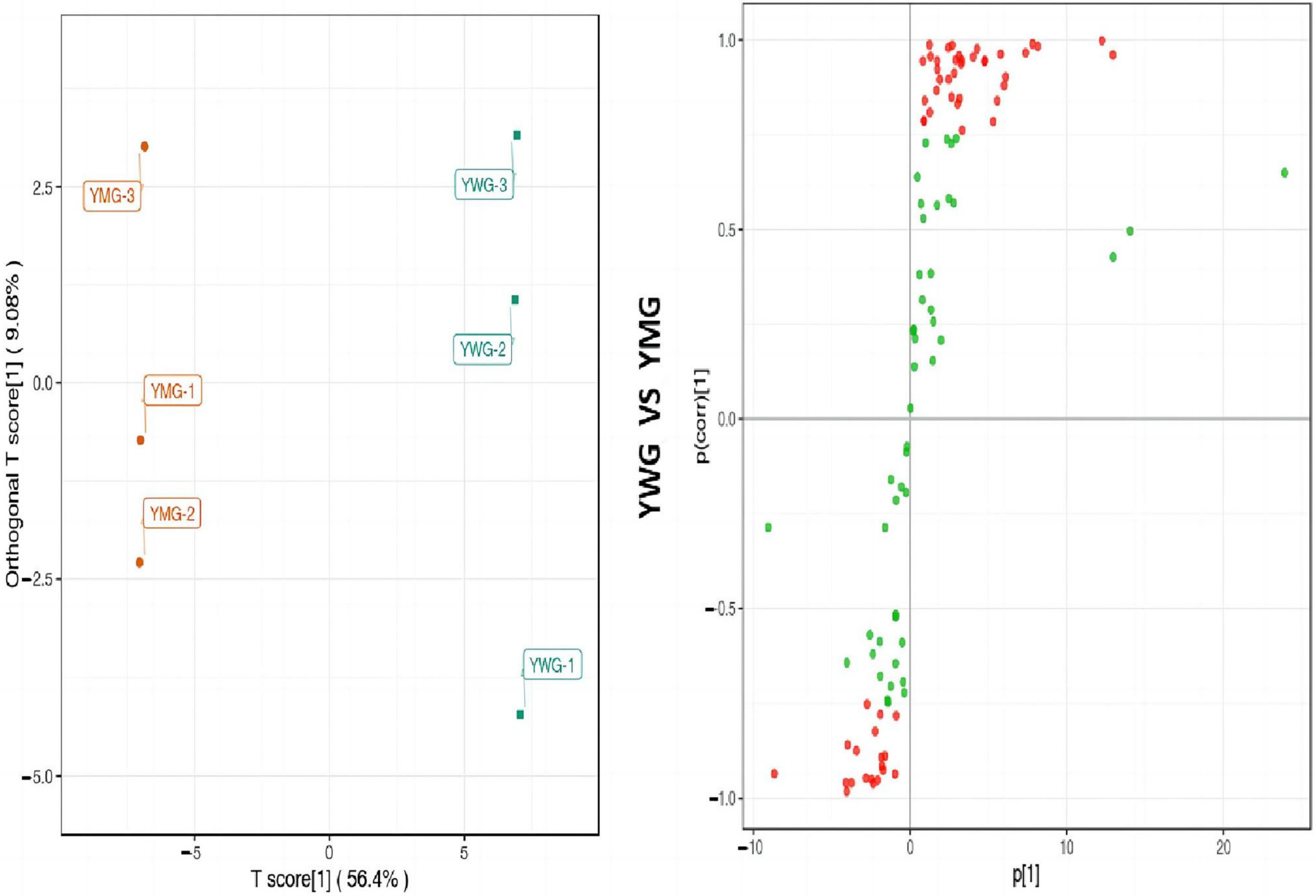

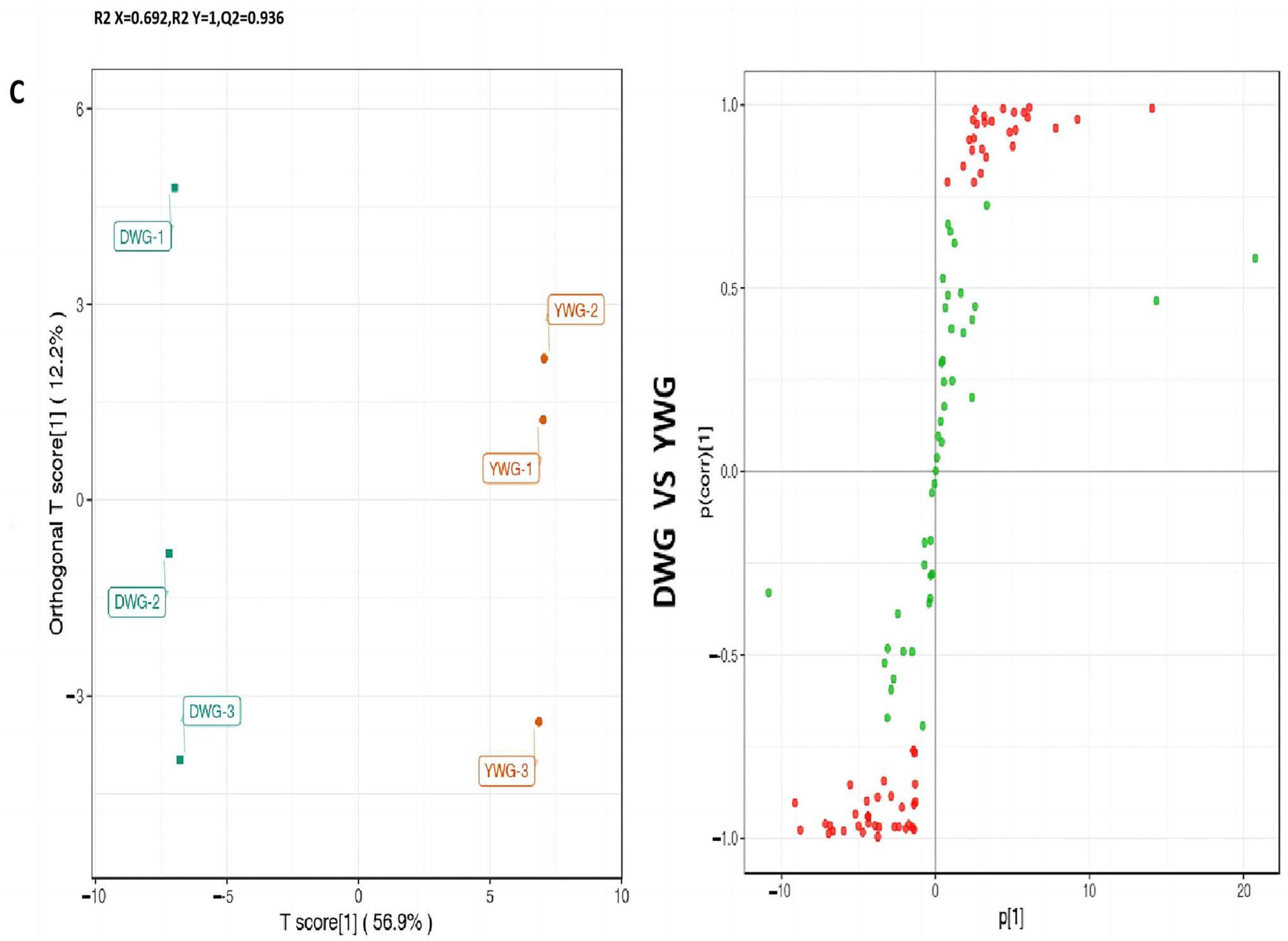
R2 X=0.692,R2 Y=1,Q2=0.936
C
DWG-1
DWG-2
DWG-3
YWG-1
YWG-2
YWG-3
Orthogonal T score[1] (12.2%)
T score[1] (56.9%)
DWG VS YWG
p(corr)[1]
p[1]

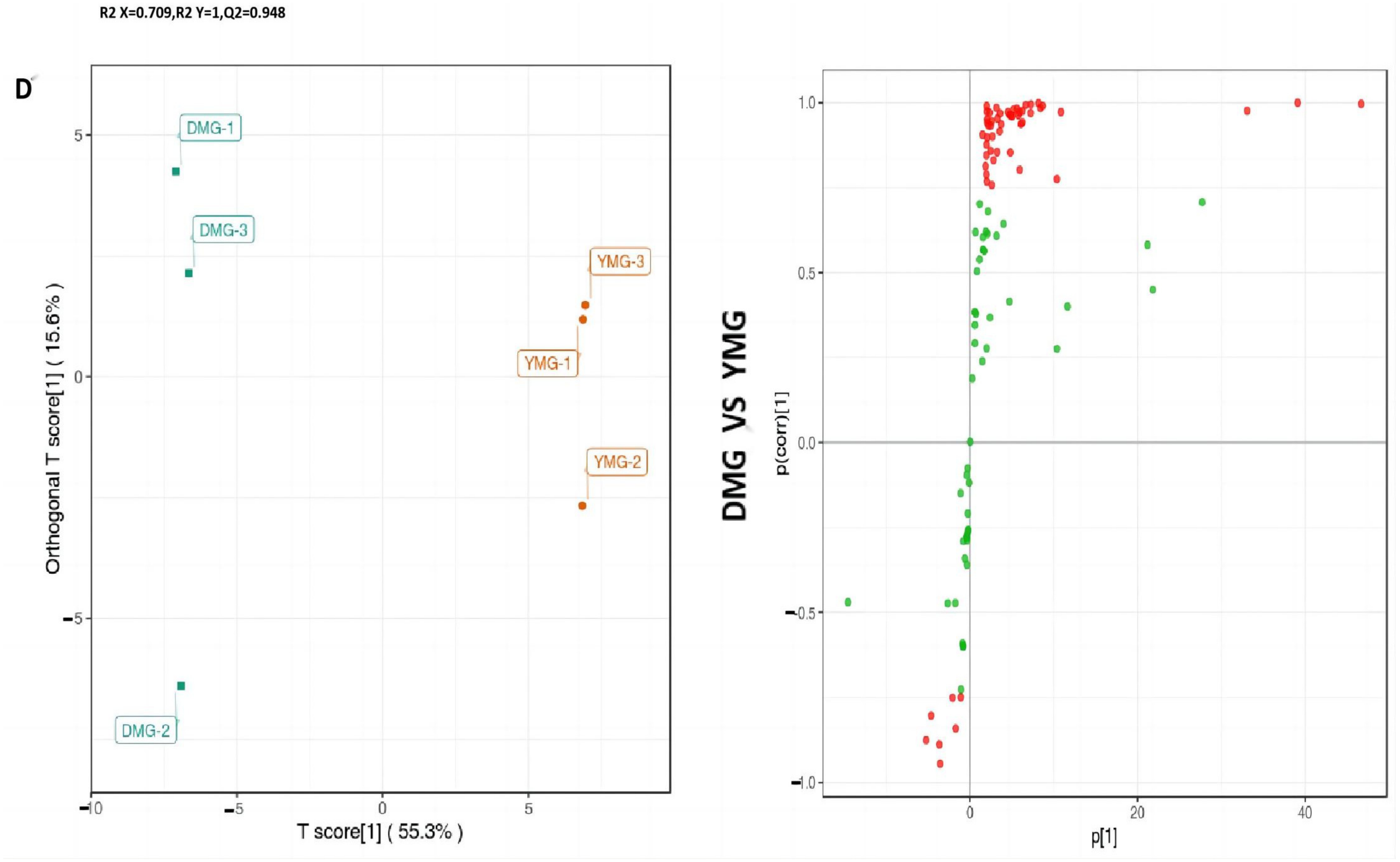

Figuse S2. The OPLS-DA score plots of D and Y between WG and MG. A-D, OPLS-DA permutation plots for the comparison groups DWG vs DMG, YWG vs YMG, DWG vs YWG, DMG vs YMG, respectively.

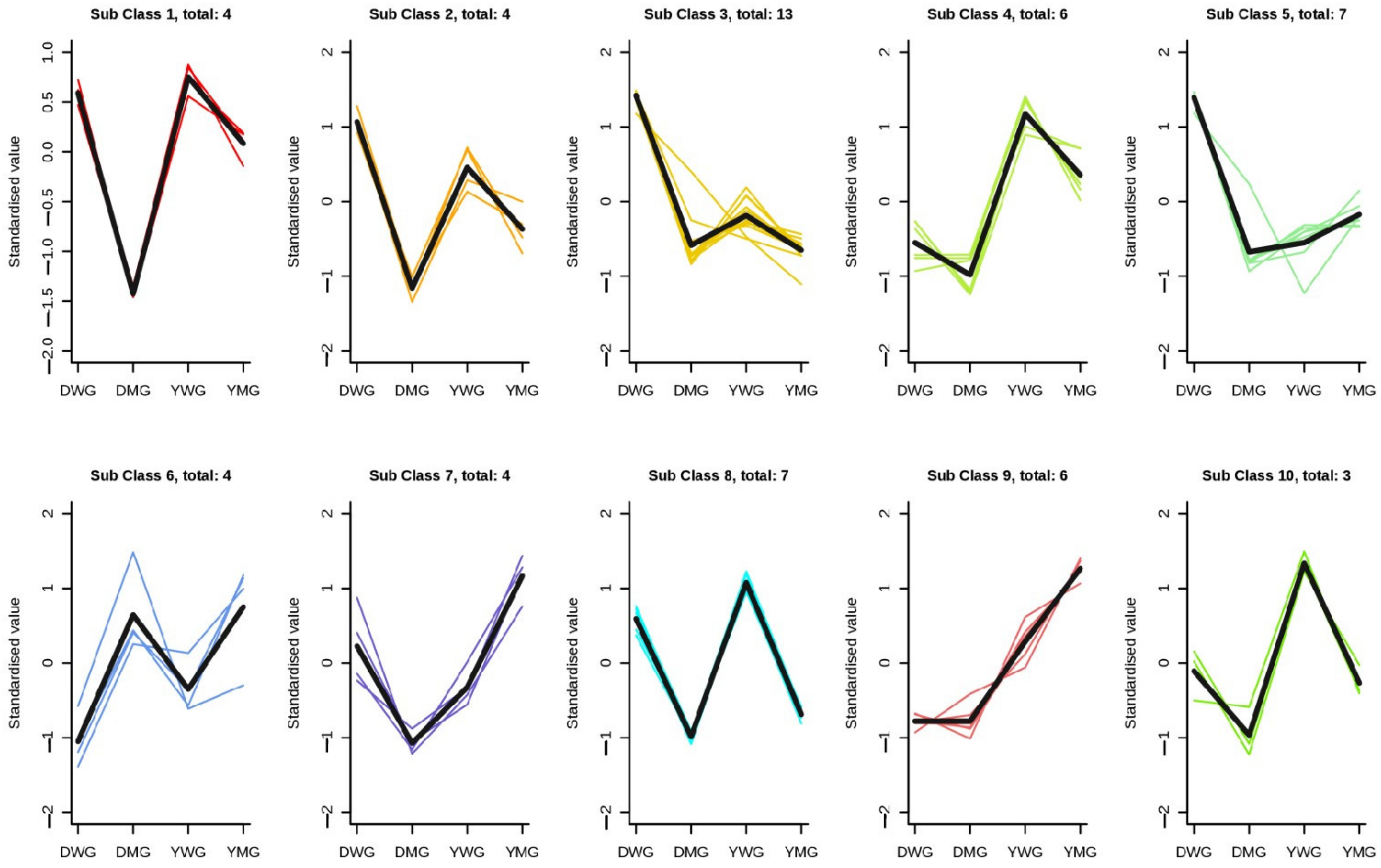

Figure S3. K-means analysis of differentially accumulated amino acids and derivative components in Yunnan hulled wheat WG and MG.

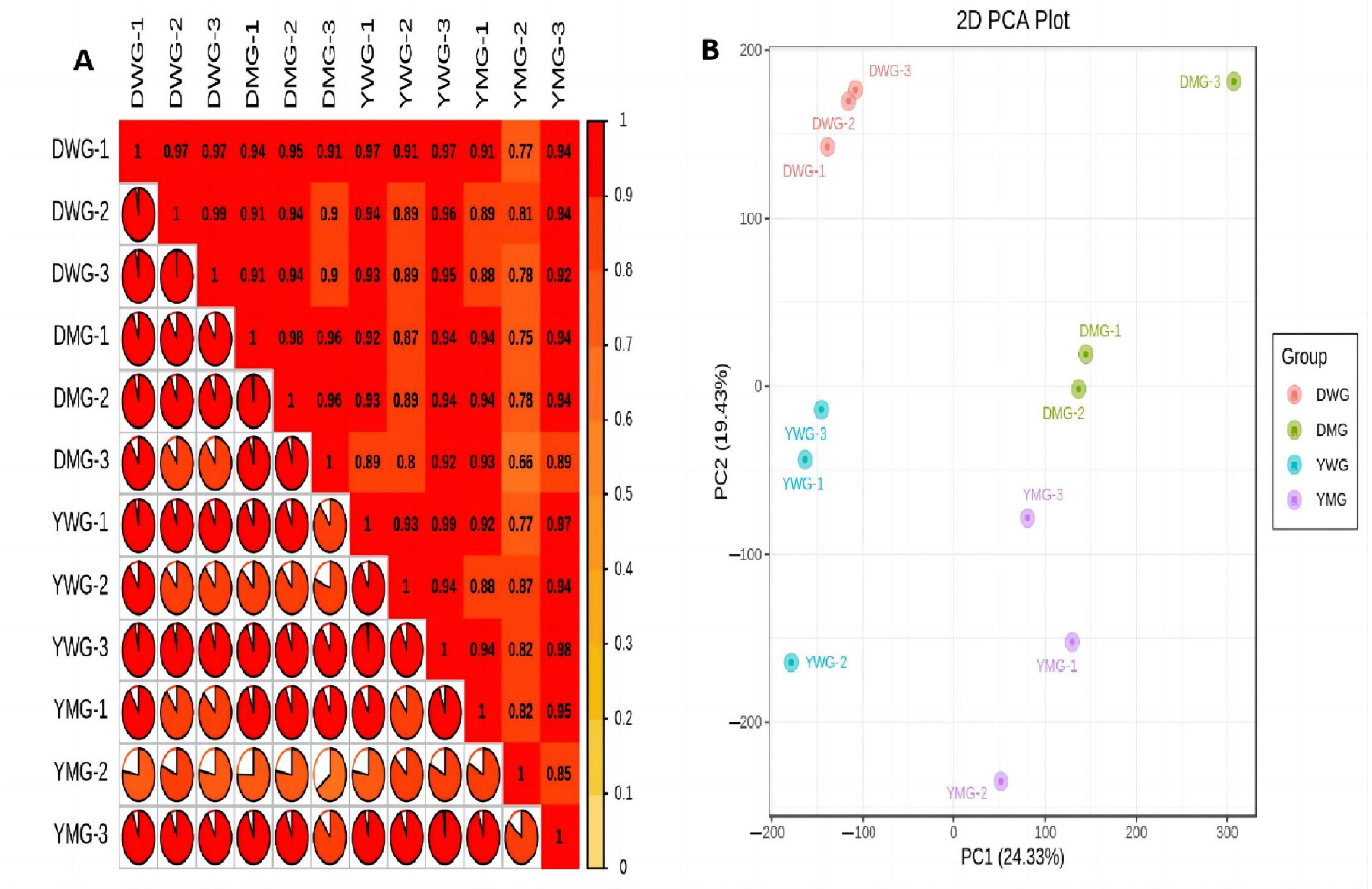

Figure S4. Correlation and principal component analysis of WG and MG samples of two Yunnan hulled wheat lines.

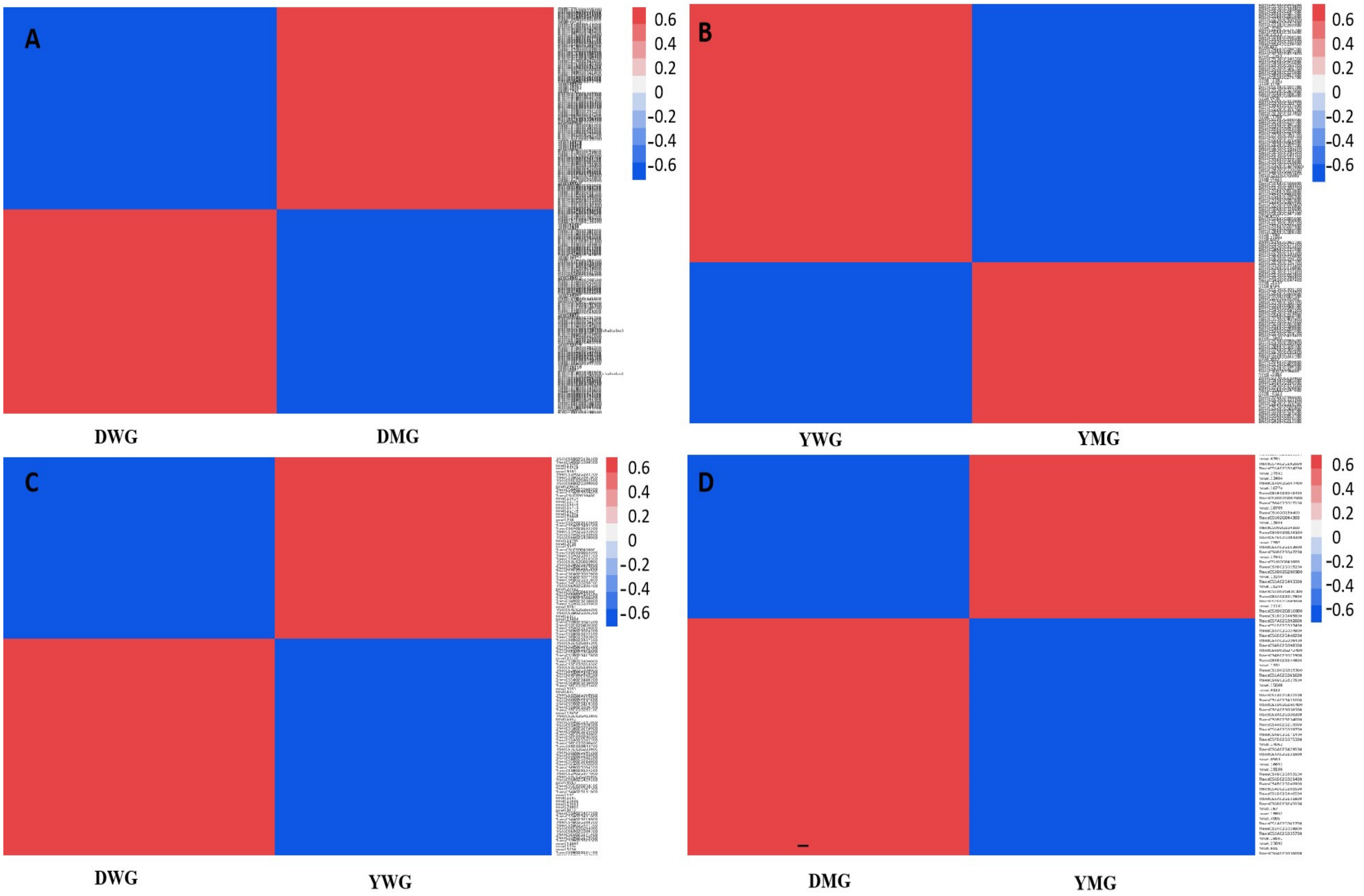

Figure S5. The different gene expression level among four comparision groups

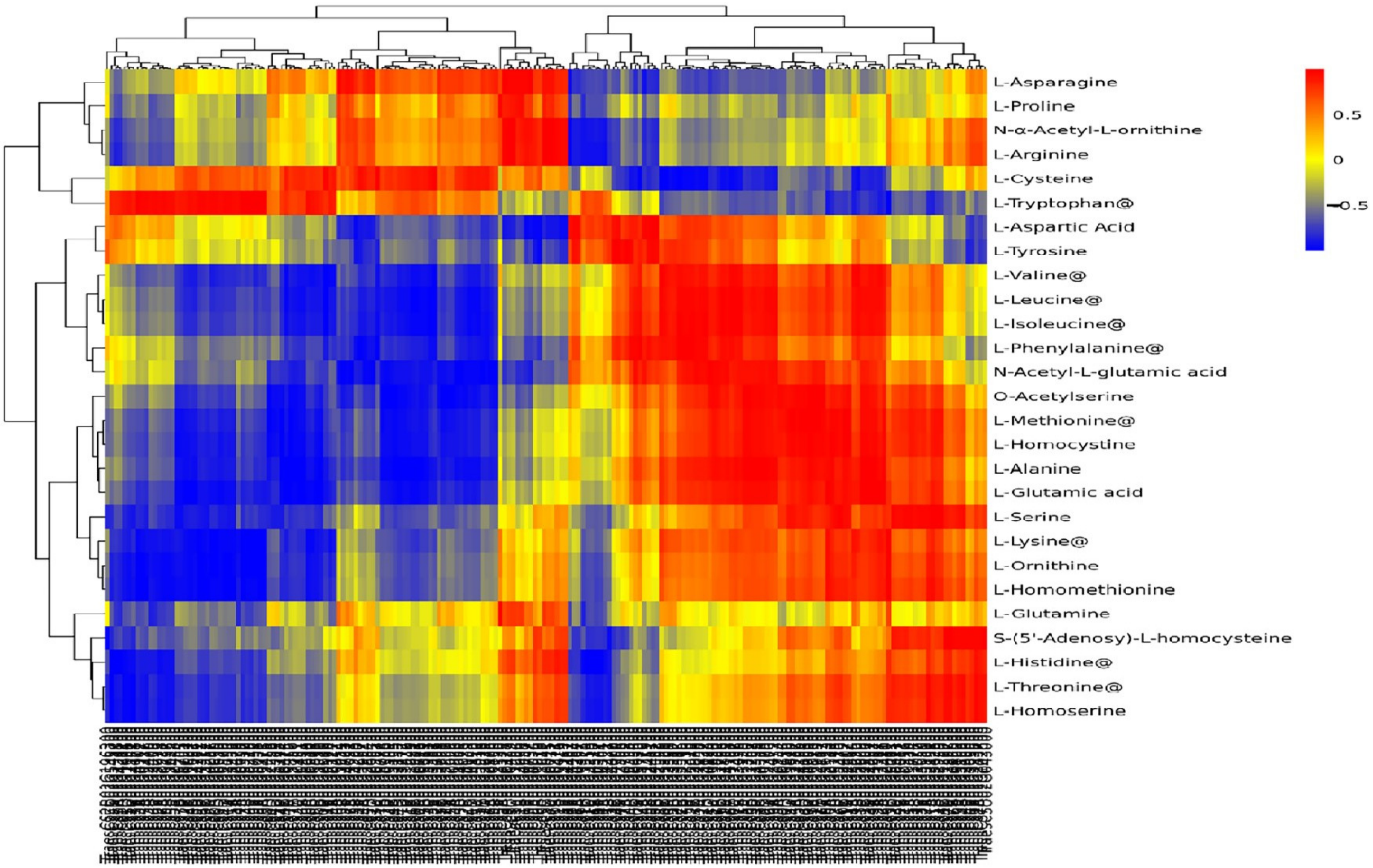

Figure S6. The correlation cluster heatmap analysis with different structured genes and key metabolites in amino acid synthesis related metabolic pathways.
